# *In situ* SAXS studies of a prototypical RAFT aqueous dispersion polymerization formulation: monitoring the evolution in copolymer morphology during polymerization-induced self-assembly†

**DOI:** 10.1039/d0sc03411h

**Published:** 2020-09-18

**Authors:** Adam Czajka, Steven P. Armes

**Affiliations:** Dainton Building Brook Hill Sheffield South Yorkshire S3 7HF UK s.p.armes@sheffield.ac.uk adam.czajka@mail.co.uk

## Abstract

Small-angle X-ray scattering (SAXS) is used to characterize the *in situ* formation of diblock copolymer spheres, worms and vesicles during reversible addition–fragmentation chain transfer (RAFT) aqueous dispersion polymerization of 2-hydroxypropyl methacrylate at 70 °C using a poly(glycerol monomethacrylate) steric stabilizer. ^1^H NMR spectroscopy indicates more than 99% HPMA conversion within 80 min, while transmission electron microscopy and dynamic light scattering studies are consistent with the final morphology being pure vesicles. Analysis of time-resolved SAXS patterns for this prototypical polymerization-induced self-assembly (PISA) formulation enables the evolution in copolymer morphology, particle diameter, mean aggregation number, solvent volume fraction, surface density of copolymer chains and their mean inter-chain separation distance at the nanoparticle surface to be monitored. Furthermore, the change in vesicle diameter and membrane thickness during the final stages of polymerization supports an ‘inward growth’ mechanism.

## Abstract 2

*In situ* small-angle X-ray scattering is used to monitor the formation of diblock copolymer spheres, worms and vesicles during reversible addition-fragmentation chain transfer (RAFT) aqueous dispersion polymerization of 2-hydroxypropyl methacrylate.

## Graphical abstract

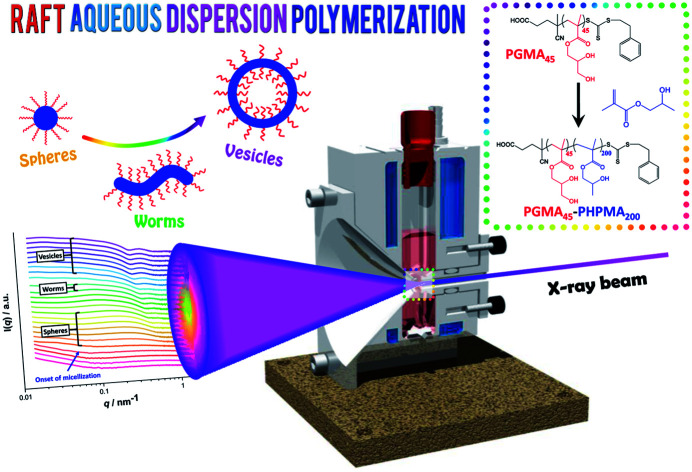

## Introduction

**Fig. 1:**
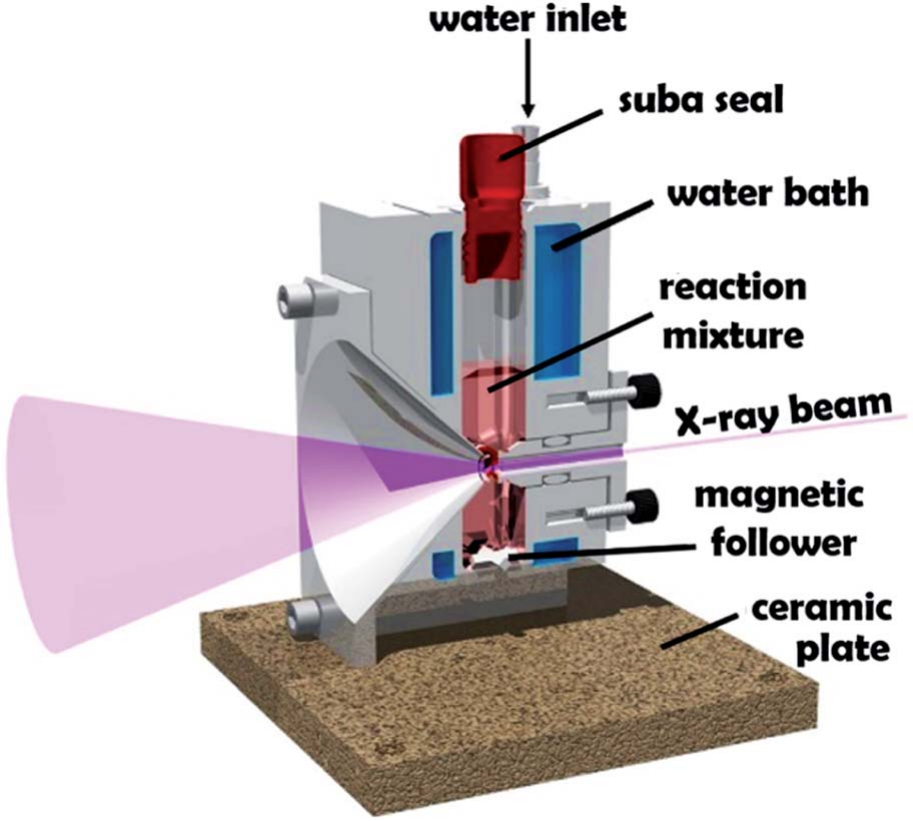
Schematic cross-section of the stirrable reaction cell used for *in situ* SAXS experiments performed during RAFT aqueous dispersion polymerization of HPMA at 70 °C. The volume of the reaction solution within this cell is approximately 2.0 mL, which is sufficient to enable *postmortem* analysis using multiple characterization techniques. Figure reprinted with permission.^61^

Block copolymer self-assembly in solution is a mature research field comprising thousands of papers dating back to the early 1960s.^1–18^ Within the last two decades, polymerization-induced self-assembly (PISA) has become widely recognized as a powerful platform technology for the efficient synthesis of a wide range of block copolymer nanoparticles of controllable size and morphology.^19–28^ In essence, PISA involves growing a second block from a soluble precursor block in a suitable solvent. As the second block grows, it becomes insoluble at some critical chain length, which leads to *in situ* self-assembly to form nascent sterically-stabilized diblock copolymer nanoparticles (or micelles). Depending on the precise PISA formulation, the final copolymer morphology is typically spheres,^29^ worms^30^ or vesicles.^15^ PISA syntheses have been conducted in water,^31^ polar solvents such as lower molecular weight alcohols,^32^ or non-polar solvents.^33^ In principle, various pseudo-living polymerization chemistries such as nitroxide-mediated radical polymerization (NMP),^34^ atom transfer radical polymerization (ATRP)^35^ or ring-opening polymerization (ROP)^36,37^ can be used for PISA. In practice, most literature reports have utilized reversible addition–fragmentation chain transfer (RAFT) polymerization.^26,38,39^ In the context of aqueous PISA syntheses, many studies have involved RAFT emulsion polymerization using water-immiscible vinyl monomers.^40–42^ However, a significant body of research has been devoted to RAFT aqueous dispersion polymerization.^43–51^ Such formulations are applicable to far fewer vinyl monomers,^52–54^ but offer a versatile route to stimulus-responsive block copolymer nano-objects,^55–57^ including highly biocompatible, readily-sterilizable thermoresponsive worm gels that can induce stasis in human stem cells.^58^ An important prototypical RAFT aqueous dispersion polymerization formulation involves the chain extension of a water-soluble poly(glycerol monomethacrylate) (GMA) precursor using 2-hydroxypropyl methacrylate (HPMA).^19^ Provided that the PGMA stabilizer block is not too long, the resulting amphiphilic PGMA-PHPMA diblock copolymer chains can form spheres, worms or vesicles depending on the target degree of polymerization for the hydrophobic structure-directing PHPMA block.^57^ When targeting PGMA_47_-PHPMA_200_ vesicles, periodic sampling of the aqueous reaction solution followed by transmission electron microscopy (TEM) studies revealed a remarkable evolution in copolymer morphology from molecularly-dissolved copolymer chains to nascent spheres, linear worms, branched worms, octopus-like and jellyfish-type intermediate structures prior to well-defined vesicles.^59^ PGMA-PHPMA vesicles display stimulus-responsive behavior,^60^ and thus offer potential for biomedical applications. Moreover, the jellyfish-type intermediates observed by TEM prior to the formation of defined PGMA-PHPMA vesicles first highlighted the possibility of *in situ* encapsulation during PISA.^59^ In principle, nanoparticles such as globular proteins (*e.g.* antibodies, enzymes, *etc.*) could be encapsulated within vesicles during their formation, with subsequent exposure to an external stimulus (*e.g.* pH or temperature) resulting in vesicle dissociation and release of the payload. Indeed, we and others recently reported the successful *in situ* encapsulation of globular proteins (bovine serum albumin, l-asparaginase),^49,62,63^ and the encapsulation and thermally-triggered release of silica nanoparticles.^64,65^ Clearly, TEM studies can provide important insights into the true nature of PISA.^59^ However, the rather poor sampling statistics and possibility of sample preparation artefacts means that such TEM images may not always be truly representative of the intermediate species. Such uncertainty also applies to cryo-TEM, which is becoming much more widely available to soft matter scientists. In principle, these limitations can be overcome by using small-angle X-ray scattering (SAXS).^66–68^ Indeed, this well-known characterization technique has already been utilized to perform *in situ* studies of the RAFT dispersion polymerization of benzyl methacrylate in non-polar media (mineral oil).^69^ These experiments required a synchrotron X-ray source and confirmed a hitherto unsuspected vesicle growth mechanism that had been previously suggested for an aqueous PISA formulation.^68^ Recently, we reported the first *in situ* SAXS studies during RAFT emulsion polymerization using a bespoke stirrable reaction cell to ensure sufficient mechanical agitation of such heterogeneous reaction mixtures (see Fig. 1).^61^ In principle, the inherently homogeneous nature of RAFT aqueous dispersion polymerization formulations should aid *in situ* SAXS studies. Very recently, Brendel and co-workers conducted *in situ* SAXS experiments to study micelle formation and growth during the RAFT aqueous dispersion polymerization of poly(*N*-acryloylmorpholine)–poly(*N*-acryloylthiomorpholine).^70^ However, their investigation focused solely on the formation of spherical nanoparticles. In the present study, we revisit a prototypical PISA formulation to conduct the first *in situ* SAXS experiments during RAFT aqueous dispersion polymerization of HPMA, for which the *in situ* evolution in copolymer morphology from spheres to worms to vesicles has already been established.^59^ We report the first detailed study of the growth of spheres as an *intermediate* morphology, the first ever *in situ* study of worm growth, and the first detailed *in situ* study of vesicle growth during aqueous PISA. This is important, because it enables the relationship between (i) the final sphere diameter and the initial worm cross-sectional diameter and (ii) the final worm cross-sectional diameter and the vesicle membrane thickness to be examined. We also explore the relationship between worm dimensions (*i.e.*, length and cross-sectional diameter) and mean aggregation number during worm growth.

## Results and discussion

### PISA synthesis protocol and kinetic data

**Fig. 2:**
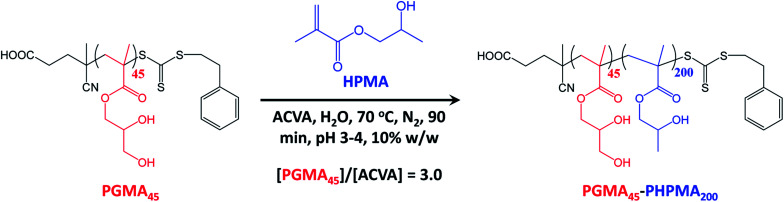
Synthesis of PGMA_45_-PHPMA_200_ diblock copolymer nano-objects *via* RAFT aqueous dispersion polymerization of HPMA using a water-soluble PGMA_45_ precursor block at 70 °C and targeting a PHPMA DP of 200.

**Fig. 3:**
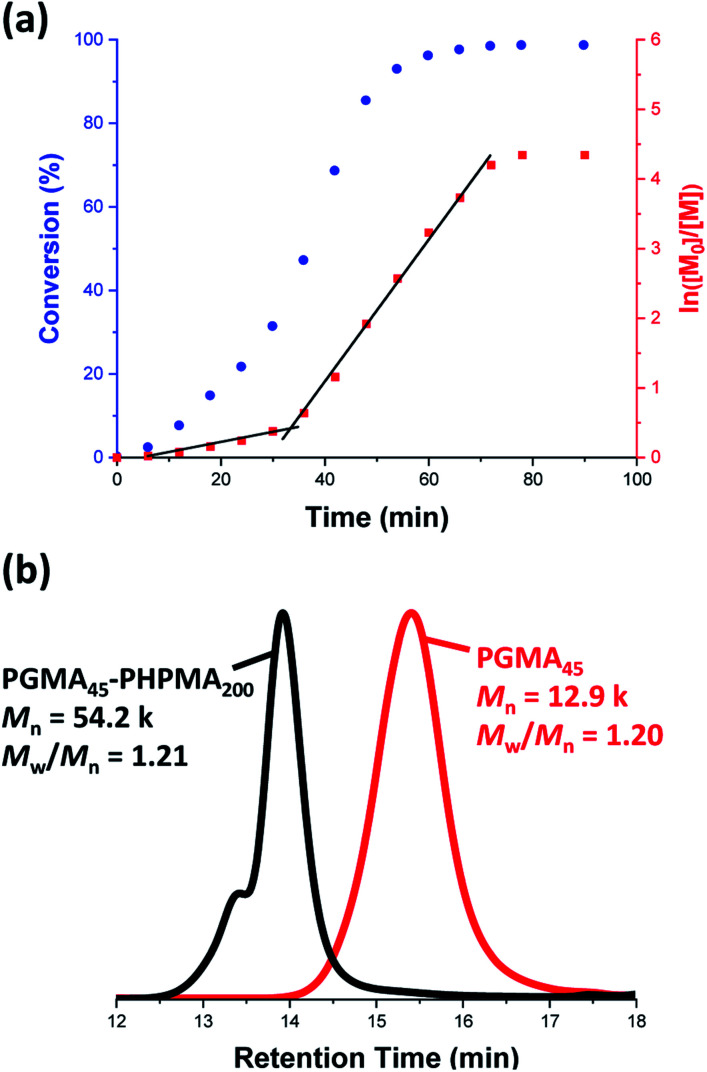
(a) Kinetic study of the laboratory-based RAFT aqueous dispersion polymerization of HPMA at 70 °C targeting PGMA_45_-PHPMA_200_ diblock copolymer vesicles at 10% w/w solids. The conversion *vs.* time data and corresponding semilogarithmic plot are denoted by blue circles and red squares, respectively. HPMA conversions were calculated from ^1^H NMR spectra recorded for quenched aliquots of the reaction solution diluted in D_2_O. (b) DMF gel permeation chromatograms recorded for the final PGMA_45_-PHPMA_200_ diblock copolymer (black curve) prepared *via* RAFT aqueous dispersion polymerization of HPMA at 70 °C at 10% w/w solids and the corresponding PGMA_45_ precursor (red curve).

A poly(glycerol monomethacrylate) (PGMA) macromolecular chain transfer agent (macro-CTA) was prepared in ethanol at 70 °C by RAFT solution polymerization of GMA using a trithiocarbonate-based RAFT agent (PETTC). The crude PGMA macro-CTA was purified by precipitation into excess dichloromethane. ^1^H NMR studies confirmed a degree of polymerization (DP) of 45 for this purified macro-CTA with an *M*_n_ of 54 200 g mol^−1^ and an *M*_w_/*M*_n_ of 1.20. This PGMA_45_ macro-CTA was then chain-extended by RAFT aqueous dispersion polymerization of 2-hydroxypropyl methacrylate (HPMA) at 70 °C at pH 3–4 to produce well-defined PGMA_45_-PHPMA_200_ diblock copolymer vesicles at 10% w/w solids, see Fig. 2. The kinetics of this HPMA polymerization were assessed by withdrawing 50 μL aliquots periodically from the reaction mixture; the polymerization was quenched by cooling to 20 °C with concomitant exposure to air. Instantaneous monomer conversions were determined by ^1^H NMR spectroscopy using sodium 2,2-dimethyl-2-silapentane-5-sulfonate (DSS) as an internal standard. More than 99% HPMA conversion was achieved within 80 min at 70 °C, see Fig. 3. Blanazs *et al.* reported a slower rate of polymerization when targeting PGMA_47_-PHPMA_200_ diblock copolymer vesicles at the same temperature, with full conversion requiring around 2 h.^59^ However, a macro-CTA/initiator molar ratio of 6 was utilized in this prior study whereas in the present study this molar ratio was reduced to 3. This difference accounts for the faster rate of polymerization observed in the present study. Moreover, a trithiocarbonate-based RAFT agent was used here instead of the dithiobenzoate-based RAFT agent employed in the prior. It is well-known that the former reagents are (i) more resistant to premature hydrolysis when used for aqueous formulations^71,72^ and (ii) less susceptible to retardation problems.^73^

Gel permeation chromatography (GPC) studies indicate that a near-monodisperse diblock copolymer was obtained with a relatively narrow molecular weight distribution (*M*_w_/*M*_n_ = 1.21), see Fig. 3.

The high molecular weight shoulder observed for the GPC trace recorded for the diblock copolymer is attributed to a small amount of dimethacrylate impurity in the HPMA monomer, which leads to light cross-linking of the PHPMA chains.^47,59^ At some critical DP, the growing PHPMA chains become sufficiently hydrophobic to form micelles. Inspecting the NMR kinetic data shown in Fig. 3, a five-fold increase in the rate of HPMA polymerization is observed after 35 min. Previously, such a rate enhancement has been attributed to micellar nucleation.^43,46^ This is because a relatively high local concentration of HPMA monomer is expected within the nascent micelles, which should lead to a microcompartmentalization effect. The instantaneous HPMA conversion after 35 min is approximately 40%, which corresponds to a critical PHPMA DP of 80.

### *In situ* SAXS studies during RAFT aqueous dispersion polymerization of PGMA_45_-PHPMA_200_

The stirrable reaction cell shown in Fig. 1 has been recently used to conduct *in situ* SAXS experiments during RAFT aqueous emulsion polymerization.^61^ However, data analysis was really rather rudimentary in this prior study.^61^ This is because the instantaneous monomer conversion at any given time point was unknown. Hence, intermediate SAXS patterns could not be fitted, which severely limited the structural information that could be extracted. Nevertheless, monitoring the change in low *q* gradient during polymerization confirmed the expected evolution in copolymer morphology from spheres to worms to vesicles, and particle dimensions were estimated from local minima for selected scattering patterns.^61^

Very recently, Brendel and co-workers reported an *in situ* SAXS study of a RAFT aqueous dispersion polymerization formulation that targeted spheres as the final phase.^70^ In contrast, the present study is the first to target vesicles with spheres as an *intermediate* morphology. This is important, because it provides an opportunity to examine the gradual evolution in copolymer morphology from dissolved chains to spheres to worms to vesicles that is believed to occur during such PISA syntheses.^59^ The details of the bespoke reaction cell have been described previously.^61^ Importantly, the cell reaction volume is around 2.0 mL, which is sufficient to enable *postmortem* characterization of the resulting diblock copolymer nanoparticles. In contrast, the much smaller sample volume (∼125 μL) of the capillaries previously employed for *in situ* SAXS studies of RAFT dispersion polymerization formulations in mineral oil^69^ preclude comprehensive analysis of the final dispersion of diblock copolymer nano-objects. To achieve the required temporal resolution for the relatively fast kinetics of polymerization (Fig. 3), a synchrotron X-ray source is essential for such *in situ* studies. This enables many high-quality SAXS patterns to be acquired over short time scales, which enables the gradual evolution in copolymer morphology during polymerization to be monitored.

**Fig. 4:**
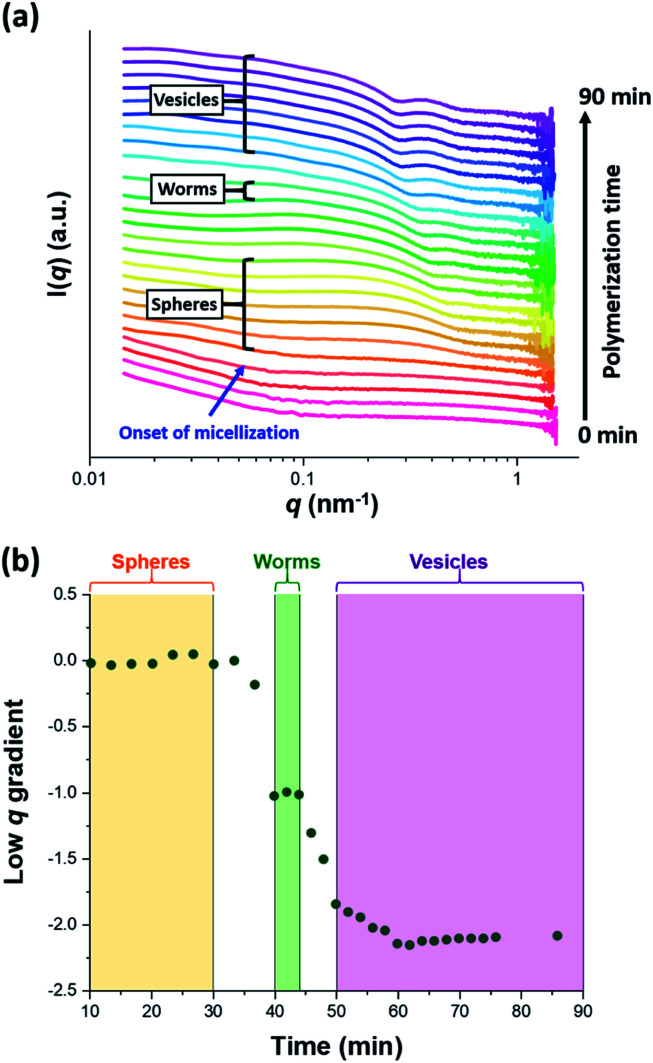
(a) SAXS patterns recorded *in situ* during the RAFT aqueous dispersion polymerization of HPMA at 70 °C targeting PGMA_45_-PHPMA_200_ vesicles at 10% w/w solids. The onset of micellar nucleation is indicated by the blue arrow. Also labelled are representative SAXS patterns corresponding to the three main copolymer morphologies (spheres, worms and vesicles) based on the low *q* gradient calculated for the 0.05 ≤ *q* ≤ 0.10 nm^−1^ region. (b) Change in this low *q* gradient during PISA synthesis: a zero gradient denotes spheres, worms are characterized by a gradient of approximately −1 and vesicles exhibit a gradient of between −2.0 and −2.5.^61,74^

The synthesis of PGMA-PHPMA diblock copolymer nano-objects *via* RAFT aqueous dispersion polymerization is a prototypical PISA formulation that has been subject to many studies.^75–77^ It has been shown to produce well-defined spheres, worms or vesicles in concentrated aqueous solution^59^ and detailed phase diagrams have been constructed for various PGMA-PHPMA diblock compositions.^57^ A particularly relevant prior study is the detailed examination of the evolution of copolymer morphology from spheres to worms to vesicles when targeting PGMA_47_-PHPMA_200_ diblock copolymer vesicles at 10% w/w solids by TEM.^59^ This diblock copolymer composition and reaction conditions closely matches those targeted in the present study, so a similar evolution in structure was anticipated.^78^Fig. 4 shows the X-ray scattering intensity, I(*q*), plotted as a function of the scattering vector, *q*, for selected SAXS patterns recorded *in situ*, as well as the gradient at low *q vs.* time during the RAFT aqueous dispersion polymerization of HPMA at 70 °C targeting PGMA_45_-PHPMA_200_ vesicles at 10% w/w solids. Scattering patterns are scaled by an arbitrary factor in Fig. 4a to improve clarity. The gradient in the low *q* regime can be used to assign the predominant morphology of the scattering objects.^74^ Spheres, rigid rods (a reasonable approximation for worms),^74^ and vesicles with relatively thin membranes (or relatively flat bilayers) exhibit low *q* gradients of approximately zero, −1 and −2, respectively, see Fig. 4b.

### *In situ* SAXS studies of the onset of micellar nucleation

Initially, the growing PGMA-PHPMA diblock copolymer chains remain soluble in the reaction mixture because the unreacted HPMA monomer acts as a co-solvent for the hydrophobic PHPMA block. The rate of solution polymerization is relatively slow during this period. According to the rate enhancement observed by ^1^H NMR spectroscopy for the equivalent laboratory-based PISA synthesis presented in Fig. 3a, the growing PHPMA chains become sufficiently hydrophobic to induce micellar nucleation on reaching a critical DP of 80 after 35 min, which corresponds to a HPMA conversion of around 40%. These observations are consistent with those made by Blanazs *et al.*, who found that the onset of micellization occurred at 46% conversion, which corresponds to a mean PHPMA DP of 90 when targeting a PGMA_47_-PHPMA_200_ formulation at 10% w/w solids.^59^ According to various studies, the onset of micellar nucleation can also be deduced by visual inspection because this event is associated with an increase in turbidity.^59,79,80^ However, visual inspection of the laboratory-based PISA synthesis of PGMA_45_-PHPMA_200_ vesicles indicated that an increase in turbidity was discernible after approximately 20 min. This suggests a somewhat earlier onset for micellar nucleation than that indicated by ^1^H NMR spectroscopy studies, for which a substantial increase in the rate of polymerization is observed after 35 min.

**Fig. 5:**
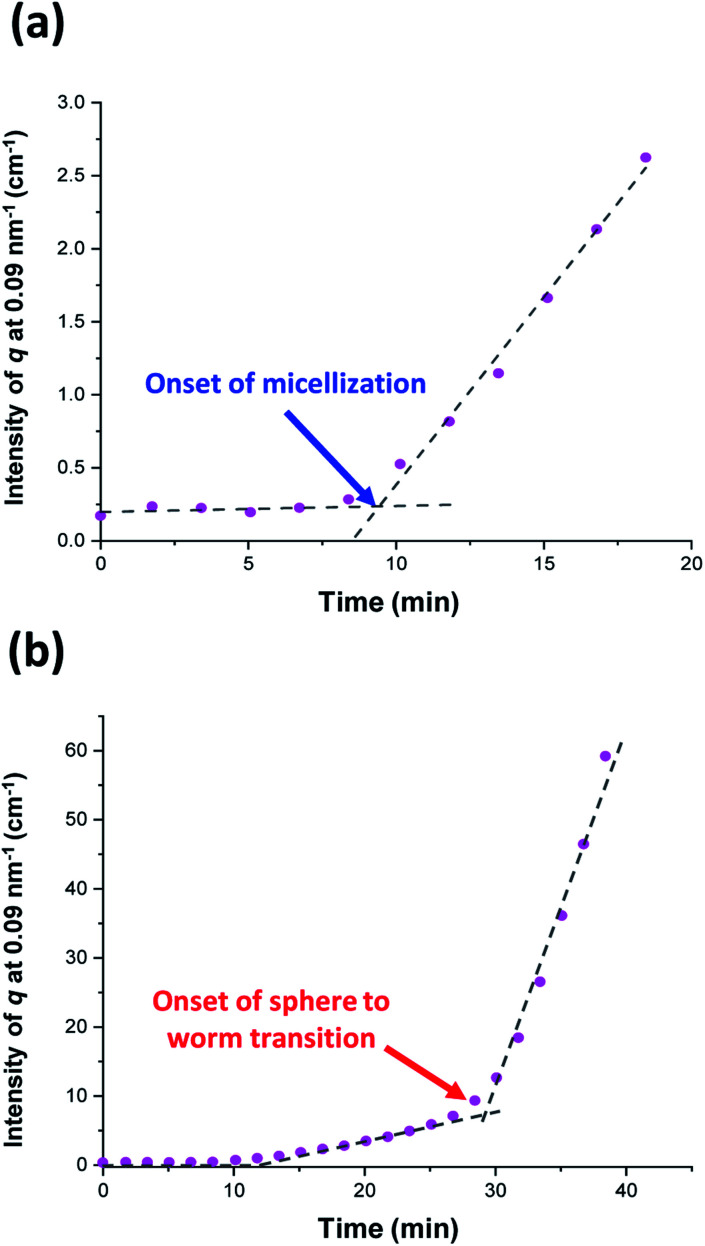
Variation in I(*q*) at an arbitrary *q* value of 0.09 nm^−1^ over time for (a) the first 20 min and (b) the first 40 min of the RAFT aqueous dispersion polymerization of HPMA at 70 °C targeting PGMA_45_-PHPMA_200_ vesicles. The blue arrow indicates the onset of micellar nucleation and the red arrow indicates the onset of the sphere to worm transition.

**Fig. 6:**
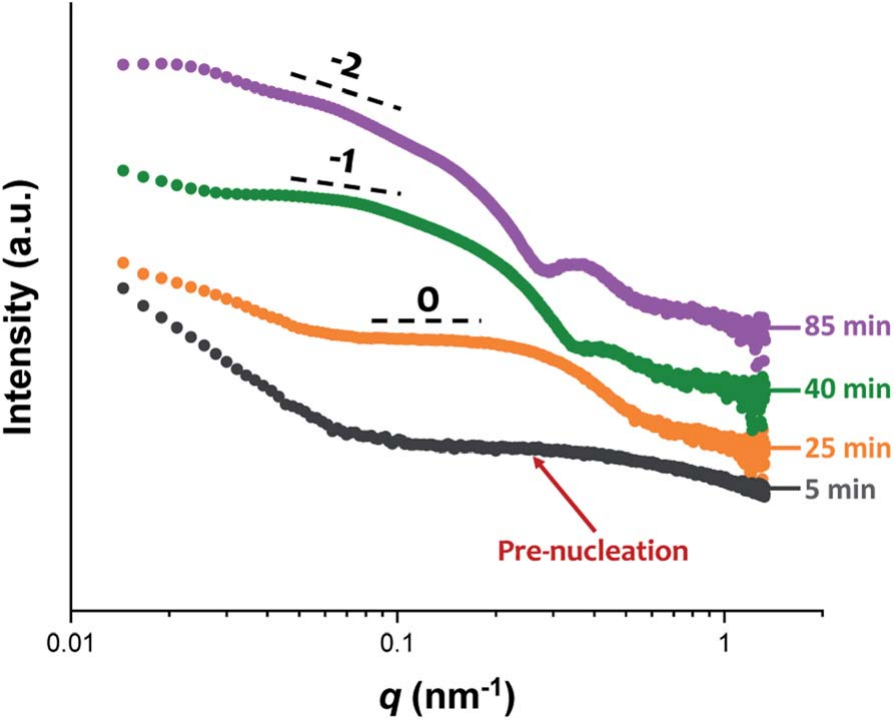
*In situ* SAXS patterns recorded during the RAFT aqueous dispersion polymerization of HPMA at 70 °C indicating the formation of spheres after 25 min, highly anisotropic worms after 40 min, and well-defined vesicles after 85 min. TEM images recorded at each of these times during the equivalent laboratory-based synthesis are consistent with these morphological assignments, see Fig. 7.

**Fig. 7:**
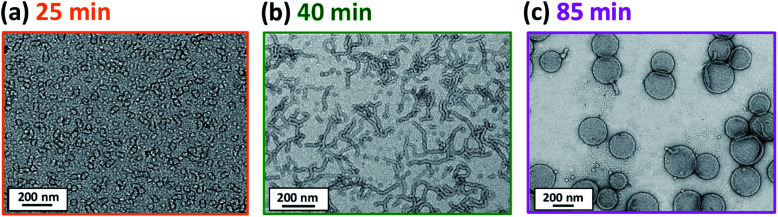
TEM images recorded at various reaction times during the laboratory-based synthesis of PGMA_45_-PHPMA_200_ vesicles illustrating the evolution in copolymer morphology with HPMA conversion: (a) spheres, (b) worms and (c) vesicles. Scattering patterns recorded at the same reaction times during the equivalent *in situ* SAXS study are consistent with these copolymer morphologies, see Fig. 6.

In principle, *in situ* SAXS can be used to determine the onset of micellization because the scattered X-ray intensity, I(*q*), is proportional to the volume of the scattering objects. Thus, micellar nucleation should be accompanied by a pronounced upturn in I(*q*).^61^ This parameter was measured at an arbitrary *q* value of 0.09 nm^−1^, see Fig. 5a. The increase in I(*q*) observed after approximately 9–10 min indicates the onset of micellar nucleation. However, this is a significantly shorter time scale compared to the rate enhancement observed by ^1^H NMR analysis for the equivalent laboratory-based PISA synthesis (35 min). According to Fig. 5b, there is a further discernible upturn in I(*q*) after approximately 28–29 min. In principle, this time point could correspond to micellar nucleation. However, scattering patterns that are characteristic of predominantly spherical objects are observed prior to 28 min, see Fig. 6. Moreover, TEM analysis of aliquots of the reaction mixture extracted during the equivalent laboratory-based PISA synthesis confirm the formation of pseudo-spherical nanoparticles within 25 min, see Fig. 7a. Thus, the pronounced upturn in I(*q*) observed after 28–29 min is instead attributed to the onset of the sphere-to-worm transition. This is physically reasonable because I(*q*) is proportional to the volume of the scattering objects, hence more intense X-ray scattering is expected for worms compared to spheres. Moreover, 1D stochastic fusion of multiple monomer-swollen spheres to form worms would lead to instantaneous access to additional unreacted HPMA monomer. Thus, this morphological transition would produce the rate enhancement indicated for the laboratory-based synthesis by ^1^H NMR studies. Furthermore, the upturn in I(*q*) at 28–29 min is reasonably consistent with the 35 min timescale observed for the *ex situ* rate enhancement. Furthermore, the scattering patterns and TEM images shown in Fig. 6 and 7 respectively are consistent with the formation of highly anisotropic worms within 40 min. These observations clearly highlight the greater sensitivity of SAXS for determining the onset of micellar nucleation during such PISA syntheses compared to TEM and ^1^H NMR spectroscopy studies.

The HPMA polymerization was judged to be complete when no further discernible change in the scattering pattern was observed. This corresponds to a reaction time of 76 min (see Fig. S3b†), which is comparable to that required for the equivalent laboratory-based synthesis (around 80 min, see Fig. 3a). In contrast, Derry *et al.* reported that the RAFT dispersion polymerization of benzyl methacrylate (BzMA) in mineral oil was complete within 120 min when targeting PSMA_31_-PBzMA_2000_ spheres during *in situ* SAXS studies at 90 °C, whereas full conversion for the equivalent laboratory-based PISA synthesis required 500 min under the same conditions.^69^ These results suggest that the choice of solvent may have a significant effect on the ability of the high-energy X-ray beam to generate an additional radical flux.^81,82^*Postmortem* DMF GPC studies indicate that the final diblock copolymer chains have a relatively narrow molecular weight distribution (*M*_n_ = 51 200 g, *M*_w_/*M*_n_ = 1.25). These data are comparable with that obtained from the equivalent laboratory-based synthesis (*M*_n_ = 54 200 g, *M*_w_/*M*_n_ = 1.21), with the respective GPC traces overlaying almost precisely, see Fig. S8.† Hence essentially the same copolymer chains are obtained in each case. Moreover, *postmortem*^1^H NMR analysis of the quenched *in situ* reaction mixture indicated a final HPMA conversion of 99%.

### *In situ* SAXS studies of PGMA_45_-PHPMA_12–73_ spheres

**Fig. 8:**
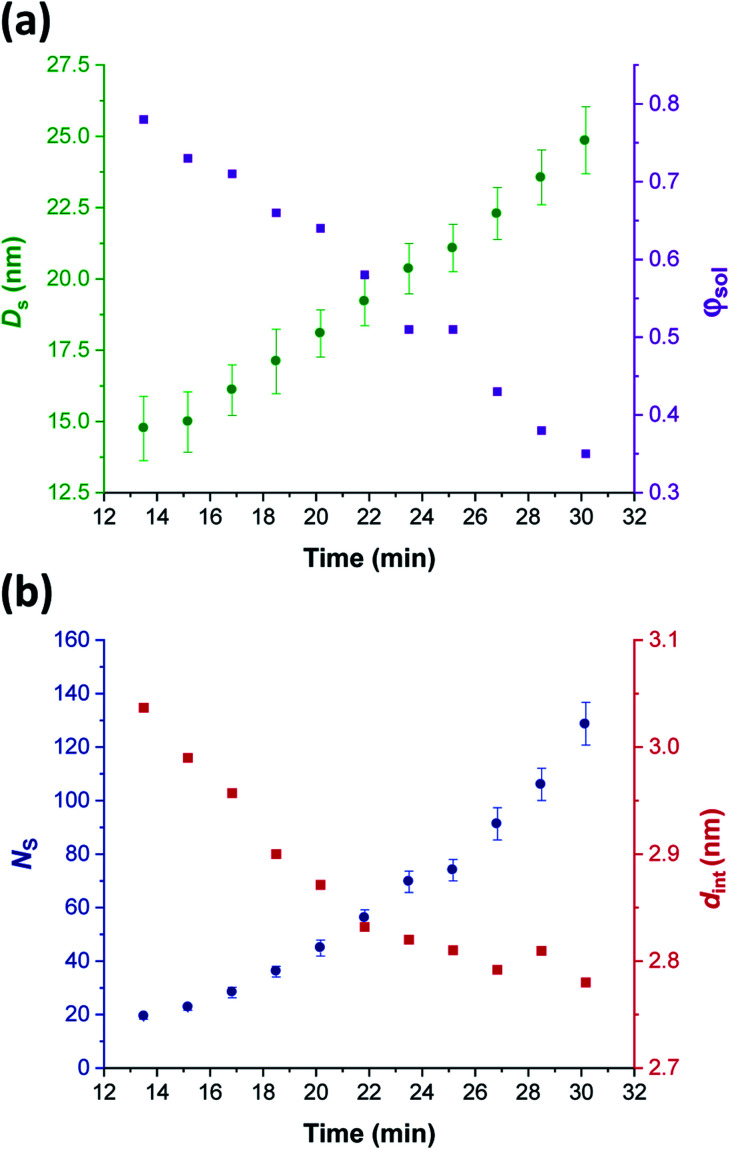
*In situ* SAXS studies of the intermediate spherical nanoparticles that are formed during the synthesis of PGMA_45_-PHPMA_200_ vesicles *via* RAFT aqueous dispersion polymerization of HPMA at 70 °C: (a) evolution in the sphere diameter (*D*_s_) and solvent volume fraction in the spherical cores (*φ*_sol_); (b) evolution in the mean number of copolymer chains per sphere (*N*_s_), and the average distance between adjacent copolymer chains at the core–shell interface (*d*_int_) [N.B. for the sake of clarity, the calculated errors for *d*_int_ values are not shown in Fig. 8 but these data are included in Table 1].

Comprehensive analysis of the *in situ* SAXS patterns recorded for diblock copolymer nano-objects requires knowledge of the instantaneous monomer conversion throughout the polymerization, because this parameter is directly related to the DP of the structure-directing block (in this case, PHPMA). Thus, HPMA conversions were calculated by renormalizing the kinetic data obtained from the laboratory-based synthesis using a sigmoid function, as reported by Derry and co-workers.^69^ The resulting conversion *vs.* time curve enables the PHPMA DP to be calculated at any time point during the PISA synthesis (see Sections 2.1 and 2.2 in the ESI†). In contrast, the structural information that could be extracted from our *in situ* SAXS study during RAFT aqueous emulsion polymerisation was much more limited as instantaneous monomer conversions were not determined in this case.^61^ X-ray scattering length densities (*ξ*_PGMA_ = 11.94 × 10^10^ cm^−2^, *ξ*_PHPMA_ = 11.11 × 10^10^ cm^−2^ and *ξ*_PH_2_O_ = 9.42 × 10^10^ cm^−2^) were calculated from the respective densities of each block as determined by helium pycnometry (*ρ*_PGMA_ = 1.31 ± 0.01 g cm^−3^ and *ρ*_PHPMA_ = 1.21 ± 0.01 g cm^−3^).^63^ Once micellar nucleation occurs, the spherical diameter (*D*_s_) of the growing nanoparticles increases more or less linearly over time, see Fig. 8a.

This parameter is calculated using the relation *D*_s_ = 2*R*_c_ + 4*R*_g_, where *R*_c_ corresponds to the mean radius of the spherical core, and *R*_g_ corresponds to the radius of gyration of the steric stabilizer block, with both radii being obtained from the spherical micelle model.^83^ The *R*_g_ for the PGMA_45_ chains was determined to be 1.81 nm, which agrees well with the estimated value of 1.71 nm (see Section 2.3 in the ESI†). The solvent volume fraction (*φ*_sol_) within the micelle cores is initially very high, but there is a two-fold reduction in this parameter as the spheres grow over time (see Table 1). This is consistent with our earlier studies, which found that longer PHPMA chains become increasingly hydrophobic.^60,84^ It is perhaps noteworthy that the substantially hydrated nature of the initial nascent micelles is likely to reduce the effective local concentration of HPMA monomer within their cores. This might explain why no rate enhancement was observed by ^1^H NMR spectroscopy after 9–10 min in Fig. 3a. However, given that so few data points were obtained within this time period, this remains an open question.

**Table 1:** Evolution of the mean degree of polymerization (DP) of the core-forming PHPMA block, the spherical nanoparticle diameter *D*_s_ (where *D*_s_ = 2*R*_s_ + 4*R*_g_), the solvent volume fraction (*φ*_sol_), mean aggregation number (*N*_s_), mean number of copolymer chains per unit surface area (*S*_agg_) and average distance between adjacent chains at the core–shell interface (*d*_int_) with increasing HPMA conversion for the intermediate spherical nanoparticles formed when targeting PGMA_45_-PHPMA_200_ vesicles *via* the RAFT aqueous dispersion polymerization of HPMA at 70 °C as determined by *in situ* SAXS studies. The standard deviation in *D*_s_ (*σ*_*D*_s__ = 2*σ*_*R*_c__) and the associated errors in *N*_s_, *S*_agg_ and *d*_int_ are also indicated

Time/min	HPMA conversion/%	PHPMA DP	*D*_s_/nm	*φ*_sol_	*N*_s_	*S*_agg_/nm^−2^	*d*_int_/nm
13.5	6.0	12	15 ± 2	0.78	19 ± 1	0.108 ± 0.007	3.04 ± 0.19
15.0	7.3	15	15 ± 2	0.73	23 ± 1	0.112 ± 0.007	2.99 ± 0.18
17.0	9.5	19	16 ± 2	0.71	28 ± 2	0.114 ± 0.007	2.96 ± 0.18
18.5	11.4	23	17 ± 2	0.66	36 ± 2	0.119 ± 0.007	2.90 ± 0.18
20.0	13.6	27	18 ± 2	0.64	45 ± 3	0.121 ± 0.007	2.87 ± 0.18
22.0	17.0	34	19 ± 2	0.58	56 ± 3	0.125 ± 0.008	2.83 ± 0.17
23.5	19.9	40	20 ± 2	0.51	70 ± 4	0.126 ± 0.008	2.82 ± 0.17
25.0	23.2	46	21 ± 2	0.51	74 ± 4	0.127 ± 0.008	2.81 ± 0.17
27.0	28.2	56	22 ± 2	0.43	91 ± 6	0.128 ± 0.008	2.79 ± 0.17
28.5	32.2	64	24 ± 2	0.38	106 ± 6	0.127 ± 0.008	2.81 ± 0.17
30.0	36.6	73	25 ± 2	0.35	128 ± 8	0.129 ± 0.008	2.78 ± 0.17

The aggregation number (or mean number of copolymer chains per sphere), *N*_s_, increases significantly during the sphere growth period, see Fig. 8b. This parameter is calculated from the estimated volume fraction of PHPMA (*φ*_PHPMA_) within the core, where *φ*_PHPMA_ = 1 − *φ*_sol_ as indicated in eqn (1).1
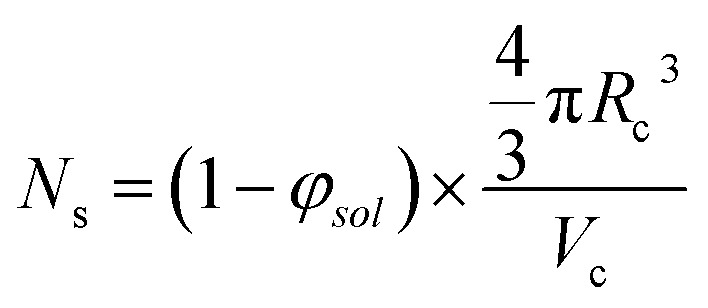


By fitting the SAXS data, the core radii (*R*_c_) were determined with relatively small experimental uncertainties. Hence the error in *N*_s_ is dominated by the associated error in the core-forming block volume (*V*_c_), see eqn (1). To estimate the maximum error in *V*_c_ at any given time during the HPMA polymerization, the molecular weight distribution determined by DMF GPC analysis of the laboratory-based synthesis (see Fig. 3b) was fitted to a Gaussian model to determine its standard deviation (see Section 2.4 of the ESI†), which was found to be approximately 6%. Having determined *N*_s_, the mean number of copolymer chains per unit surface area (*S*_agg_), and the average distance between adjacent copolymer chains at the core–shell interface (*d*_int_) can be calculated using eqn (2) and (3) respectively.2
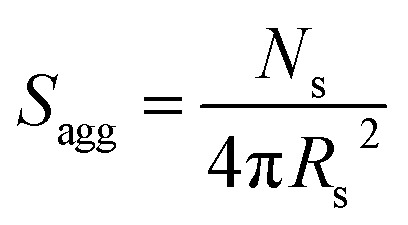
3
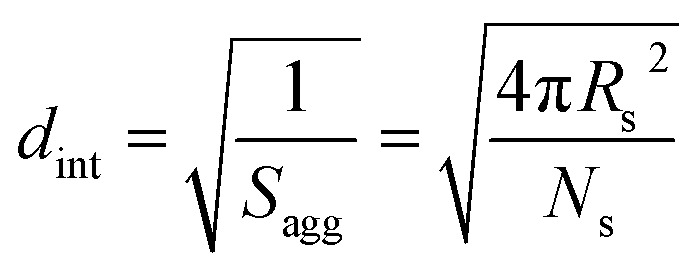


Immediately after micellar nucleation, the spheres begin to grow in size. In principle, this could occur solely as a result of the longer PHPMA chains, with the polymerization proceeding with no further change in the mean aggregation number, *N*_s_. However, Fig. 8b indicates a substantial concomitant increase in *N*_s_, which is the main reason for the observed increase in sphere size. This suggests that either sphere–sphere fusion and/or exchange of individual copolymer chains between spheres must also occur.^85^ During this time period, the spheres are highly hydrated (see Table 1). They must also be monomer-swollen, otherwise PISA cannot proceed. Such solvation most likely provides sufficient mobility for chain expulsion and thus copolymer exchange.^86,87^ Given that 1D sphere–sphere fusion is reported to be required for worm formation,^88^ it seems reasonable to postulate that 3D sphere–sphere fusion contributes to sphere growth. We also note that exchange of copolymer chains is expected to become less likely as the HPMA polymerization proceeds and the degree of core hydration of the spheres is reduced (see Table 1). Clearly, delineating the relative contributions of these sphere growth mechanisms warrants further work, but this is beyond the scope of the present study.

As *N*_s_ increases, the copolymer chains within the growing spherical nanoparticles become more closely packed together as the sphere cores gradually become more dehydrated (see Table 1). Inspecting Fig. 8b, both *S*_agg_ and *d*_int_ tend towards limiting values during the HPMA polymerization. This suggests that there is an optimum packing efficiency for the copolymer chains within the growing spherical nanoparticles.^85^

### *In situ* SAXS studies of PGMA_45_-PHPMA_134–154_ worms

Following nucleation, only pseudo-spherical micelles are initially present. Then short worms begin to form after approximately 30 min (37% conversion; PHPMA_73_), as confirmed by both the upturn in I(*q*) (see Fig. 5b) and TEM studies (see Fig. S5a†). At first, spheres and worms are in co-existence. However, relatively few spheres are present after approximately 40 min (66% conversion; PHPMA_134_). Comparing the mean aggregation numbers for the final spheres with the worms formed at this time point suggests that each worm comprises approximately 1450 ÷ 128 = 11 spheres. Subsequently, the corresponding SAXS patterns exhibit a low *q* gradient of approximately −1 for a short period (40–44 min), which indicates the formation of increasingly anisotropic worms (see Fig. 4b).^83^ Relatively long and/or branched worms constitute the primary morphology at this point, see TEM image in Fig. S5b.† This compares well with observations made by Blanazs *et al.*, who reported that worms were the primary morphology for a very similar intermediate diblock copolymer composition (PGMA_47_-PHPMA_131_).^57^ Fitting the scattering patterns recorded during this relatively short time period using an established worm-like micelle model^83^ enables the mean worm length (*L*_w_) and aggregation number (*N*_w_) to be determined. Inspecting the *in situ* SAXS data summarized in Table 2, the worm cross-sectional diameter (*D*_w_) remains more or less constant at 24.7–25.7 nm, which is essentially the same as the final mean sphere diameter (*D*_s_ = 25 nm after 30 min). However, the mean worm length increases by a factor of approximately three over the 40–44 min time period and there is a comparable increase in *N*_w_. These observations are fully consistent with a worm growth mechanism based on the stochastic 1D fusion of multiple spheres.^59,84,88^ To the best of our knowledge, this is the first detailed study of the growth of spheres and worms as *intermediate* morphologies during PISA. This is important, because it provides an opportunity to compare the relative dimensions of such nano-objects during the sphere-to-worm (and worm-to-vesicle) transition. On attaining a certain critical length, the worms begin to form branches.^83^ The number of branch points gradually increases and worm clustering begins to occur (see Fig. S5c in the ESI†). This is consistent with the abrupt increase in mean aggregation number (*N*_w_) observed between 43 and 44 min, see Table 2.

**Table 2:** Evolution of the mean degree of polymerization (DP) of the core-forming PHPMA block, the worm cross-sectional diameter (*D*_w_ = 2*R*_w_ + 4*R*_g_), mean worm length (*L*_w_), and aggregation number (*N*_w_) with increasing HPMA conversion for the intermediate worms formed when targeting PGMA_45_-PHPMA_200_ vesicles *via* the RAFT aqueous dispersion polymerization of HPMA at 70 °C as determined by *in situ* SAXS studies. The standard deviation in *D*_w_ (*σ*_*D*_w__ = 2*σ*_*R*_w__) and the associated error in *N*_w_ are also indicated

Time/min	HPMA conversion/%	PHPMA DP	*D*_w_/nm	*L*_w_/nm	*N*_w_
40	67	134	24.7 ± 1	241	1450 ± 90
41	70	140	25.0 ± 1	259	1530 ± 90
42	73	145	25.1 ± 2	316	1760 ± 110
43	75	150	25.5 ± 2	452	2470 ± 150
44	77	154	25.7 ± 2	776	4390 ± 270

Blanazs *et al.* have proposed a likely mechanism for the structural evolution from worms to vesicles for a PGMA_47_-PHPMA_200_ PISA formulation on the basis of TEM studies.^59^ Briefly, the highly branched worms and/or worm clusters undergo partial coalescence to generate octopus-like structures (see Fig. S6a†), which then wrap up to form ‘jellyfish’-type structures (see Fig. S6b†). These transient jellyfish structures are eventually transformed into well-defined vesicles (see following section).

### *In situ* SAXS studies of PGMA_45_-PHPMA_176–200_ vesicles

The scattering profiles shown in Fig. 4 begin to exhibit a low *q* gradient of approximately −2 after 50 min (88% conversion; PHPMA_176_). This feature corresponds to bilayer formation and hence indicates the presence of vesicles.^61,74^ Furthermore, a local minimum at *q* ≈ 0.30 nm^−1^ associated with the vesicle membrane thickness also becomes discernible. Accordingly, scattering patterns recorded after this time point were fitted using a polydisperse vesicle model.^11^ This enabled various structural parameters such as the vesicle diameter (*D*_v_), vesicle membrane thickness (*T*_m_), and mean aggregation number (*N*_v_) to be determined, see Table 3.

**Table 3:** Evolution of the mean degree of polymerization (DP) of the core-forming PHPMA block, the vesicle diameter (*D*_v_ = 2*R*_out_ + 4*R*_g_), membrane thickness (*T*_m_), solvent volume fraction associated with the weakly hydrophobic chains (*φ*_sol_) and mean vesicle aggregation number (*N*_v_) with increasing HPMA conversion, as determined by *in situ* SAXS analysis during the latter stages of the PISA synthesis of PGMA_45_-PHPMA_200_ vesicles *via* RAFT aqueous dispersion polymerization of HPMA at 70 °C. The standard deviation in *D*_v_ (*σ*_*D*_v__ = 2*σ*_*R*_v__) and the associated errors in *T*_m_ and *N*_v_ are also indicated

Time/min	HPMA conversion/%	PHPMA DP	*D*_v_/nm	*T*_m_/nm	*φ*_sol_	*N*_v_
50	88	176	215 ± 14	14.2 ± 2	0.38	29 700 ± 1800
54	92	185	221 ± 16	15.2 ± 2	0.40	32 300 ± 2000
56	93	188	226 ± 16	15.7 ± 2	0.39	34 900 ± 2100
58	95	190	227 ± 20	16.2 ± 2	0.38	35 100 ± 2100
62	97	194	227 ± 17	16.7 ± 2	0.39	34 000 ± 2000
66	98	196	227 ± 16	16.9 ± 2	0.40	33 400 ± 2000
70	99	198	226 ± 18	16.9 ± 2	0.40	33 100 ± 2000
76	100	200	227 ± 16	17.0 ± 2	0.41	32 500 ± 2000
86	100	200	227 ± 17	17.1 ± 2	0.41	32 500 ± 2000

**Fig. 9:**
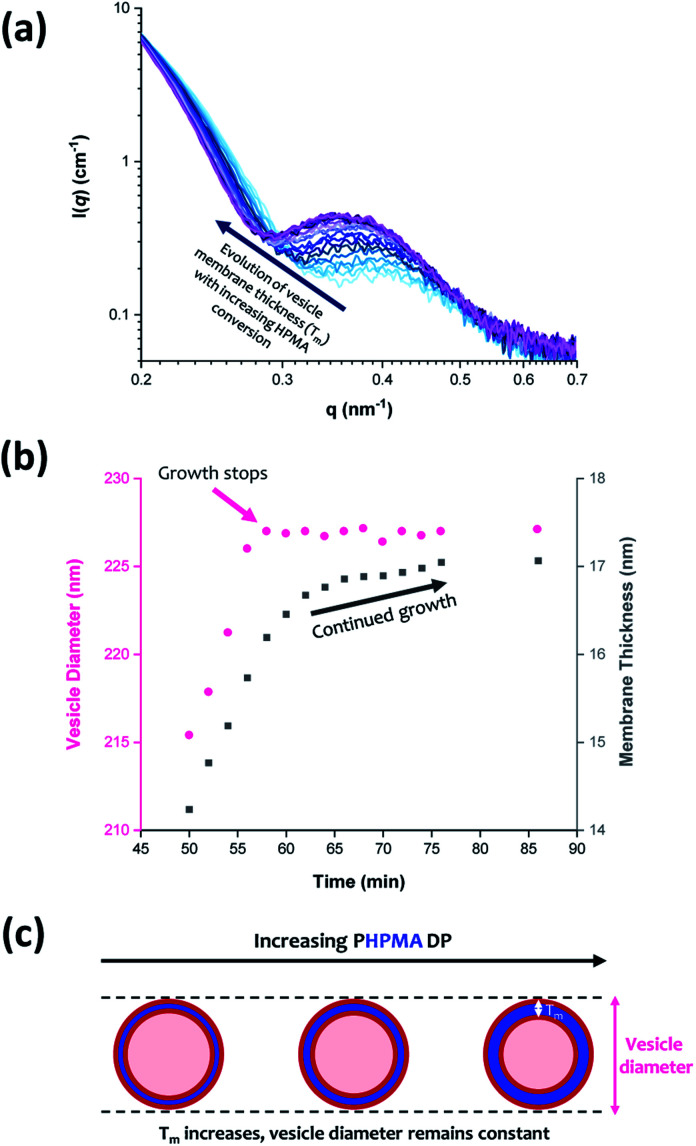
(a) *In situ* SAXS patterns recorded when targeting PGMA_45_-PHPMA_200_ vesicles *via* RAFT aqueous dispersion polymerization of HPMA at 70 °C. (b) Growth in vesicle diameter (pink circles) and membrane thickness (grey squares) over time calculated from the SAXS data shown in (a). (c) Schematic representation of the ‘vesicle inward growth’ mechanism indicated by the data shown in (b), illustrating the gradual increase in membrane thickness as the vesicle diameter remains relatively constant [N.B. the relative change in the vesicle membrane thickness is exaggerated for clarity].

From SAXS analysis, the mean membrane thickness of the initial vesicles is significantly smaller than the final worm cross-sectional diameter. This is important, because it suggests that there is substantial interdigitation of the PHPMA chains within the vesicle membranes.^89^ Moreover, the overall vesicle diameter remains essentially unchanged at approximately 227 nm after 56 min (93% conversion; PHPMA_188_). The local minimum at *q* ≈ 0.30 nm^−1^ (corresponding to the vesicle membrane thickness, *T*_m_) gradually shifts to lower *q* during the HPMA polymerization, see Fig. 9a. Thus, there is a period where the overall vesicle diameter remains constant while the vesicle membranes continue to thicken, which suggests an ‘inward growth’ mechanism during the final stages of the HPMA polymerization, see Fig. 9c. Similar observations were reported by Derry and co-workers when targeting PSMA_13_-PBzMA_150_ vesicles.^69^ It is perhaps worth emphasizing that this ‘inward growth’ mechanism differs from the ‘vesicle fusion’ mechanism reported by Eisenberg and co-workers for the evolution of the vesicle morphology during traditional post-polymerization processing using a (slow) solvent switch.^90,91^ Simple geometric considerations suggest that such a vesicle growth mechanism enables minimization of the free energy of the system.^68^ After 76 min, there was no discernible difference between consecutive scattering patterns, as highlighted by the constant intensity at *q* = 0.09 nm^−1^, see Fig. S3b.† Moreover, the various structural parameters remained relatively constant, so the HPMA polymerization is assumed to be complete at this time point.

Inspecting Table 3, there is an apparent gradual reduction in *N*_v_ from 35 100 to 32 500 during the vesicle growth stage of the PISA synthesis (*i.e.*, from 56 to 86 min). Warren *et al.* suggested that copolymer chains are expelled to relieve the growing steric congestion within the vesicle membrane during this growth stage. However, the modest reduction in *N*_v_ shown in Table 3 is within experimental error, so this explanation is considered to be unlikely for the present formulation. The number of copolymer chains per unit area (*S*_agg_) increases by 0.010 nm^−2^ over the 46 to 76 min time interval, suggesting a higher packing density owing to the inward growth of the vesicle membranes. In this case, eqn (4)–(6) are required to calculate *N*_v_, *S*_agg_ and *d*_int_ for vesicles (see Section 2.5 of the ESI† for a full description of the various parameters). TEM studies were conducted during vesicle growth but analysis of these images provided no additional evidence for membrane thickening. However, this is not unexpected, given the rather modest increase in the mean membrane thickness (*ca.* 4 nm).4
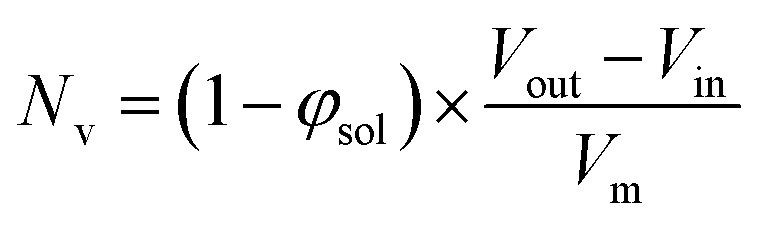
5
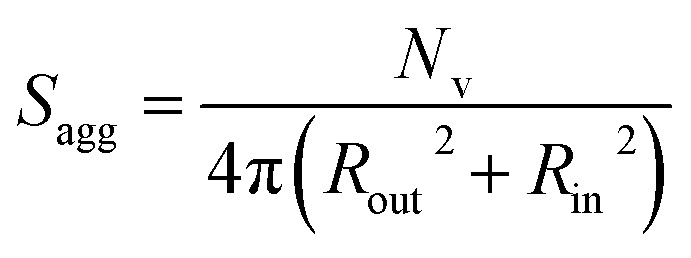
6
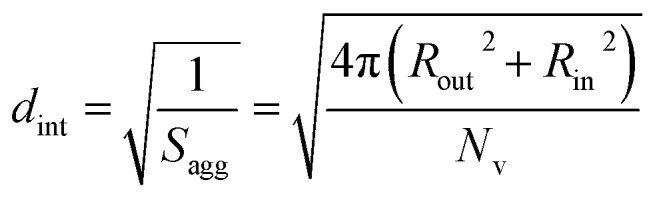


Previous reports have suggested that *T*_m_ should increase according to the power law *T*_m_ = *kx*^*α*^ where *k* is a constant and *x* is the PHPMA DP.^92,93^ A value of *α* = 0.50 indicates collapsed coils while *α* = 1.00 corresponds to fully stretched chains (*e.g.*, for *n*-alkyl chains within phospholipid liposomes). For the PGMA_45_-PHPMA_200_ vesicles reported herein, the *α* exponent was calculated to be 0.84, which is consistent with that reported by both Derry and co-workers^69^ and also Warren *et al.*^68^ Interestingly, the worm cross-sectional diameter (*D*_w_) is significantly greater than *T*_m_, which provides strong evidence for interdigitation of the structure-directing PHPMA chains within the vesicle membranes. The solvent volume fraction (*φ*_sol_) within the vesicle membranes remains relatively constant at approximately 0.40 throughout the vesicle growth period. This is rather similar to the *φ*_sol_ values observed during the latter stages of formation of the intermediate spheres (see Table 1 and Fig. 8a). These data compare well with those reported by Warren *et al.* who used SAXS to calculate a *φ*_sol_ of approximately 0.38 for PGMA_55_-PHPMA_200_ vesicles.^68^ These findings indicate a relatively high degree of hydration for the water-insoluble structure-directing PHPMA block after micellar nucleation has occurred. This is not unexpected given its weakly hydrophobic nature: variable temperature ^1^H NMR studies performed by Blanazs *et al.* indicate an even higher degree of hydration at sub-ambient temperatures.^94^ No doubt the morphological transitions that occur during this PISA synthesis are facilitated by such hydration, although it seems likely that solvation of the growing PHPMA chains by unreacted HPMA monomer also plays an important role.

Once polymerization is complete, SAXS analysis indicates a final vesicle diameter *D*_v_ of 227 ± 16 nm. Dynamic light scattering (DLS) analysis indicates an intensity-average diameter of 254 ± 9 nm (polydispersity = 0.183), while TEM analysis suggests a number-average vesicle diameter of approximately 247 nm (see Fig. S7†). Bearing in mind the effect of polydispersity, vesicle diameters are reasonably self-consistent. They also compare well with literature data reported for similar PGMA_55_-PHPMA_200_ vesicles, for which SAXS studies indicated a *D*_v_ of 244 ± 5 nm.^68^^1^H NMR spectroscopy analysis suggests essentially full monomer conversion (>99%) within similar timescales for the *in situ* SAXS study and the laboratory-based synthesis. Moreover, *postmortem* DMF GPC studies indicate that the final diblock copolymer chains exhibit a relatively narrow molecular weight distribution (*M*_n_ = 51 200; *M*_w_/*M*_n_ = 1.25). These data are consistent with that obtained from the equivalent laboratory-based synthesis (*M*_n_ = 54 200; *M*_w_/*M*_n_ = 1.21), see Fig. S8.† These observations suggest that RAFT aqueous dispersion polymerization syntheses conducted using the bespoke stirrable reaction cell are essentially identical to those performed under normal laboratory conditions.

## Conclusions

The RAFT aqueous dispersion polymerization of HPMA at 70 °C leads to the formation of well-defined vesicles when using a suitable PGMA_45_ precursor as a steric stabilizer block and targeting a PHPMA DP of 200. The *in situ* evolution in copolymer morphology from dissolved chains to spheres to worms to vesicles for this prototypical PISA formulation can be conveniently monitored by *in situ* SAXS studies using a stirrable reaction cell. The volume of the reaction solution within this cell is approximately 2.0 mL, which is sufficient to enable *postmortem* analysis of the final vesicle dispersion using ^1^H NMR spectroscopy, DLS and TEM, as well as GPC analysis of the final PGMA_45_-PHPMA_200_ diblock copolymer chains.

*In situ* SAXS studies indicate that micellar nucleation occurs within 9–10 min. Once nucleation has occurred, spherical nanoparticles grow in size over time, with a gradual reduction in the degree of hydration of the hydrophobic PHPMA cores and a concomitant increase in the mean aggregation number. The number of copolymer chains per unit area and the inter-chain separation distance both reach limiting values for the growing spheres, which suggests an optimum packing efficiency. The first short worms are formed at a PHPMA DP of around 134. Subsequently, the mean worm cross-sectional diameter remains essentially constant while the worm length and mean aggregation number both increase rapidly *via* stochastic 1D fusion of multiple spheres. Prior TEM studies suggest that the worms then begin to form branch points, eventually fusing together to form vesicles *via* transient ‘jellyfish’ intermediate structures.^59^ The initial vesicle membrane thickness is significantly less than the final worm cross-sectional diameter, which indicates substantial interdigitation of the PHPMA chains. During the final stages of the HPMA polymerization, the vesicle membrane (*T*_m_) becomes progressively thicker as the overall vesicle dimensions remain almost constant, hence the lumen volume gradually shrinks. This vesicle growth mechanism reduces the total interfacial area and hence allows minimization of the free energy of the system.^68,69^ SAXS analysis indicates a final vesicle diameter (*D*_v_) of 227 ± 16 nm, which is consistent with *postmortem* DLS and TEM studies. Furthermore, excellent agreement is obtained when comparing *postmortem* GPC data with the equivalent laboratory-based synthesis, which indicates that essentially the same copolymer chains are generated in each case. Clearly, *in situ* SAXS can provide important insights into the true nature of PISA. This is a key characterization technique for developing our fundamental understanding, which should guide the future design of diblock copolymer nano-objects for various potential applications. Moreover, our findings are expected to be of considerable interest to theoreticians, who are beginning to turn their attention towards modelling PISA formulations.^95–97^

## Conflicts of interest

There are no conflicts to declare.

## Supplementary Material

SC-011-D0SC03411H-s001
