# Supplementary material for: In situ SAXS studies of a prototypical RAFT aqueous dispersion polymerization formulation: monitoring the evolution in copolymer morphology during polymerization-induced self-assembly
Source: Chem Sci. 2020 Sep 18;11(42):11443–54. doi: 10.1039/d0sc03411h (PMC8162469; doi:10.1039/d0sc03411h)
Supplement: SC-011-D0SC03411H-s001 [file SC-011-D0SC03411H-s001.pdf]

Electronic Supplementary Material (ESI) for Chemical Science.
This journal is © The Royal Society of Chemistry 2020

Supporting Information for:

In situ SAXS Studies of a Prototypical RAFT Aqueous Dispersion Polymerization Formulation: Monitoring the Evolution in Copolymer Morphology during Polymerization-Induced Self-Assembly

Adam Czajka* and Steven P. Armes*

*Dainton Building, Department of Chemistry, University of Sheffield, Brook Hill, Sheffield, South Yorkshire, S3 7HF, UK

Table of contents

1) Experimental Section S2
1.1) Polymer Characterization. S3

2) Supporting Analysis S5
2.1) Determining theoretical DPs S5
2.2) Calculating individual block volumes S6
2.3) Estimating stabilizer chain length (R_g) S6
2.4) Maximum error in mean aggregation number S7
2.5) N_{agg}, S_{agg}, and d_{int} equations S8

3) Supporting Figures S9
3.1) Micellar Nucleation S9
3.2) Data fits to selected SAXS data S10
3.3) TEM image of spheres, worms and nascent vesicles S11
3.4) TEM images of intermediate structures S12
3.5) TEM image of the final vesicles S13
3.6) Comparison of GPC data for the lab-based synthesis and *in situ* SAXS experiment . S14

4) SAXS Models S15
4.1) Spherical micelle model S15
4.2) Worm-like micelle model S17
4.3) Vesicle model S18

1. Experimental Section

Materials

The following materials were used without further purification unless stated.

Glycerol monomethacrylate (GMA; GEO Specialty Chemicals, 99%), 2-hydroxypropyl methacrylate (HPMA; GEO Specialty Chemicals, 97%), 4,4'-azobis(4-cyanopentanoic acid) [ACVA] (Aldrich, 99%), 4-cyano-4-((2-phenylethanesulfanyl)thiocarbonylsulfanyl)-pentanoic [PETTC] (prepared in-house following a previously described protocol[1]), methanol (Aldrich, 99%), anhydrous ethanol (Aldrich, 99%), dichloromethane (Aldrich, 99%), sodium 2,2 dimethyl-2-silapentane-5-sulfonate (DSS; Aldrich), D_2O (Cambridge Isotopes, 99.9%), CD_3OD (Cambridge Isotopes, 99.9%). Deionized water was obtained from an Elga DV25 water purifier unit.

Synthesis of $PGMA_{45}$ macro-CTA

PETTC RAFT agent (0.90 mmol, 0.30 g), GMA monomer (44.2 mmol, 7.08 g), ACVA (0.20 mmol, 49.8 mg, CTA/ACVA molar ratio = 5.0) and anhydrous ethanol (11.14 g, 40% w/w) were weighed into a 50 mL round-bottomed flask, placed in an ice bath and purged with N_2 for 30 min. The sealed flask was immersed into an oil bath set at 70 °C and stirred for 120 min (GMA conversion = 76%, as determined by ^{1}H NMR studies in CD_3OD). After 120 min, the reaction mixture was diluted with methanol (25 mL) and precipitated into excess dichloromethane (twice). The precipitate was dissolved in deionized water and any residual dichloromethane was removed under reduced pressure. The purified product was freeze-dried overnight to afford a PGMA macro-CTA as a yellow powder. ^{1}H NMR (CD_3OD) indicated a mean degree of polymerization of 45 for this precursor. GPC analysis (DMF eluent, refractive index detector, series of near-monodisperse poly(methyl methacrylate) calibration standards) indicated an M_n of 12 900 g mol^{-1} and a M_w/M_n = 1.20.

Kinetic studies of the RAFT Aqueous Dispersion Polymerization of HPMA at 70 °C when targeting $PGMA_{45}$-$PHPMA_{200}$ vesicles

$PGMA_{45}$ macro-CTA (49.9 mg, 6.61 µmol), HPMA monomer (0.191 g, 1.32 mmol; target DP = 200), DSS (5.77 mg, 26.4 µmol, HPMA/DSS molar ratio = 50), and ACVA (0.60 mg, 2.20 µmol, CTA/ACVA molar ratio = 3.0) were weighed into a 10 mL round-bottomed flask and purged with N_2 gas for 20 min. Deionized water (2.18 mL, 10% w/w formulation), separately degassed with N_2 for 30 min, was then added. A sample was immediately taken for ^{1}H NMR analysis, and the reaction solution was degassed for a further 5 min prior to immersion in an oil bath set at 70 °C. The 'zero time' (t = 0 min) for this polymerization was arbitrarily taken to be the point when the degassed reaction solution was first immersed in the 70 °C oil bath, rather than the time at which the reaction solution had reached this temperature. Aliquots were subsequently removed under N_2 via syringe at various time intervals for ^{1}H NMR, DLS and

TEM analysis. Each aliquot was quenched by dilution in D_2O at 20 °C. Monomer conversions were normalized using the DSS as an internal standard and are expressed relative to the HPMA/DSS molar ratio observed at 'zero time'. For both TEM and DLS analysis, aliquots were diluted fifty-fold with deionized water at 20 °C to produce 0.20% w/w dispersions.

In situ SAXS studies of the RAFT Aqueous Dispersion Polymerization of HPMA at 70 °C when targeting PGMA$_{45}$-PHPMA$_{200}$ vesicles

PGMA$_{45}$ macro-CTA (50.0 mg, 6.63 µmol), HPMA monomer (0.191 g, 1.33 mmol; target DP = 200), ACVA (0.60 mg, 2.21 µmol, CTA/ACVA molar ratio = 3.0) and deionized water (2.17 mL, 10% w/w formulation) were weighed into a 14 mL sample vial and purged with N_2 for 30 min. This reaction solution was then transferred via degassed syringe to the SAXS cell (containing a magnetic flea and equipped with a magnetic stirrer unit) which had been separately purged with N_2 for 20 min. Once the SAXS cell was attached to the sample stage in I22 and aligned relative to the SAXS beam, the HPMA polymerization was initiated using a water-circulating jacket to heat the cell up to 70 °C as the X-ray beam shutter was opened. The polymerization was monitored until no further evolution in the 1D SAXS pattern was observed, at which point it was assumed that the reaction was complete.

1.1 Polymer Characterization

^{1}H NMR Spectroscopy

All NMR spectra were recorded in either D_2O or CD_3OD using a 400 MHz Bruker Avance-400 spectrometer (64 scans averaged per spectrum).

Gel Permeation Chromatography (GPC)

Copolymer molecular weights and dispersities were determined using a DMF GPC setup operating at 60 °C and comprising two Polymer Laboratories PL gel 5 µm mixed-C columns connected in series to a Varian 390-LC multidetector suite (refractive index detector only) and a Varian 290-LC pump injection module. The GPC eluent was HPLC-grade DMF containing 10 mM LiBr at a flow rate of 1.0 mL min^{-1} and DMSO was used as a flow-rate marker. Calibration was conducted using a series of ten near-monodisperse poly(methyl methacrylate) standards (M_n = 625 to 618,000 g mol^{-1}). Chromatograms were analyzed using Varian Cirrus GPC software (version 3.3).

Dynamic Light Scattering (DLS)

DLS studies were conducted on 0.20% w/w aqueous dispersions at 25 °C in disposable cuvettes using a Malvern Zetasizer NanoZS instrument, which detects back-scattered light at

an angle of 173°. Intensity-average hydrodynamic diameters were calculated via the Stokes−Einstein equation using a non-negative least-squares (NNLS) algorithm. All data were averaged over three consecutive runs.

Transmission Electron Microscopy (TEM)

Removed aliquots were diluted fifty-fold at 20 °C to generate 0.20% w/w dispersions. Copper/palladium TEM grids (Agar Scientific, UK) were surface-coated in-house to yield a thin film of amorphous carbon. The grids were then plasma glow-discharged for 30 s to create a hydrophilic surface. Individual samples (0.20% w/w, 5 µL) were adsorbed onto the freshly glow-discharged grids for 1 min and then blotted with filter paper to remove excess solution. To stain the aggregates, uranyl formate solution (0.75 %w/v, 5.0 µL) was soaked on the sample-loaded grid for 20 seconds and then carefully blotted to remove excess stain. The grids were then dried using a vacuum hose. Imaging was performed on a Technai T12 Spirit instrument operating at 120 kV and equipped with a Gatan 1 k CCD camera.

Small-Angle X-ray Scattering (SAXS)

SAXS patterns were recorded at an international synchrotron facility (station ID02 at ESRF, Grenoble, France). A monochromatic X-ray beam (λ = 0.0995 nm) and a 2D SAXS detector (Rayonix MX-170HS) were used for these experiments. A q range of 0.004−2.0 nm^{-1} was used for all measurements, where $q = (4\pi \sin \theta)/\lambda$, corresponds to the modulus of the scattering vector and θ is half of the scattering angle. A bespoke stirrable reaction cell was used for these time-resolved measurements, see Figure 1 in the main text. SAXS patterns were recorded every 10 seconds for the first 10 min, every 30 seconds for the next 30 min and every 60 seconds thereafter until no further change in the SAXS patterns could be observed. X-ray scattering data were reduced (integrated, normalized, and background-subtracted) using standard routines available at the ID02 beamline. The scattering intensity of water was used for absolute scale calibration of the X-ray scattering patterns. Irena SAS macros for Igor Pro were utilized for modelling and further SAXS analysis.

2. Supporting Analysis

2.1 Determining Theoretical DPs

Laboratory-based kinetic data were normalized and fitted to a sigmoid function, see Figure S1.

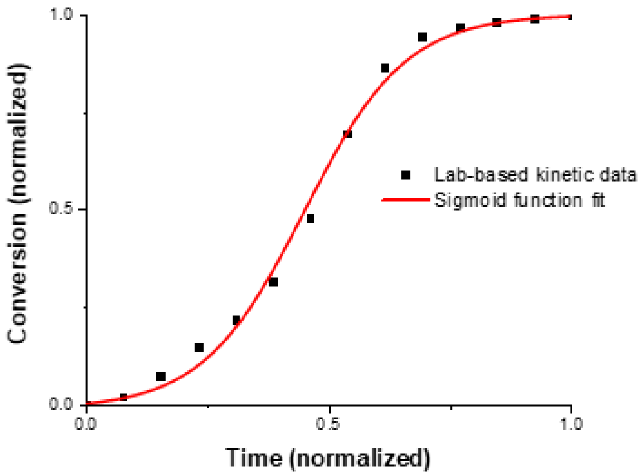

Figure S1. Renormalization of the polymerization kinetics data obtained by ^{1}H NMR spectroscopy. Laboratory-based kinetic data are shown as black squares. Red line corresponds to the sigmoid function fit to these data. Both time and conversion have been normalized.

The sigmoid function shown in Figure S1 is described by the following equation:

$$y = a + \frac{b}{\left(1 + exp\left(\frac{c - x}{d}\right)\right)} \quad \text{(S1)}$$

where y is the HPMA conversion, x is the relative polymerization time and *a*, *b*, *c* and *d* are arbitrary fitting parameters.

For the sigmoid function fit shown in Figure S1, the values for *a*, *b*, *c* and *d* are -0.01, 1.01, 0.44965 and 0.10469, respectively. Thus y can be calculated for any value of x. For example, 42 min (72% conversion) corresponds to a theoretical PHPMA DP of 145, see below [N.B. The relative polymerization time (x) was normalized by dividing each time point by 76 min, which corresponds to the overall reaction time].

$$y = -0.01 + \frac{1.01}{\left(1 + exp\left(\frac{0.44965 - (\frac{42}{76})}{0.10469}\right)\right)} = 0.725$$

Now the calculated value of y is simply multiplied by the final DP of the core-forming PHPMA block. For this reaction, ^{1}H NMR spectroscopy studies indicated essentially full conversion, so the PHPMA DP = 200. Hence the calculated DP after 42 min is given by:

$$y = 0.725 \times 200 = 145$$

2.2 Calculating Individual Block Volumes

To analyze the scattering patterns reported in this study, the scattering models employed require knowledge of both the core and stabilizer block volumes. To calculate the instantaneous DP for the core-forming block, see Section 2.1 above. Armes with this DP, individual block volumes can be calculated using Equation S2:

$$V_{block} = \frac{MW \; x \; DP}{\rho \; x \; N_a} \quad \text{(S2)}$$

where MW corresponds to the monomer molecular weight, DP is the mean degree of polymerization of the block, ρ is the density and N_a is Avogadro's constant. The respective mass densities for PGMA and PHPMA are ρ_{PHPMA} = 1.21 ± 0.01 g cm^{-3} and ρ_{PGMA} = 1.31 ± 0.01 g cm^{-3}, as determined in a previous study using helium pycnometry.[2]

Following Section 2.1, the instantaneous PHPMA DP corresponds to 145 after 42 min. This DP can be converted into a corresponding PHPMA block volume using Equation S2 (see below):

$$V_{\text{PHPMA}} = \frac{144.17 \text{ g mol}^{-1} \text{ x } 145}{1.21 \text{ g cm}^{3} \text{ x } 6.022 \text{ x } 10^{23} \text{ mol}^{-1}} \text{ x } (1 \text{ x } 10^{24})$$

(conversion to Å^3)

$$V_{\text{PHPMA}} = 28\ 689 \text{ Å}^3$$

2.3 Estimating the Stabilizer Chain Length (*R*g)

A $PGMA_{45}$ precursor was employed as the steric stabilizer in this study. The experimental R_g for this precursor in aqueous solution was calculated to be 1.81 nm by fitting its SAXS pattern to a Gaussian chain model. The contour length of such $PGMA_{45}$ chains can be estimated by assuming each monomer repeat unit comprises two carbon bonds in an all *trans* configuration, which equates to a segment length of 0.255 nm. Thus the contour length of a $PGMA_{45}$ chain, $L_{PGMA_{45}}$ = 45 x 0.255 nm, or 11.475 nm. Given a mean Kuhn length of 1.53 nm (based on the known literature value of PMMA[3]) the radius of gyration, R_g, can be estimated to be:

$$R_g = (11.475 \text{ x } \frac{1.53}{6})^{0.5} = 1.71 \text{ nm}$$

This estimated R_g agrees well with that obtained experimentally from SAXS fitting suggesting that it is a physically reasonable value.

2.4 Maximum error in the mean aggregation number

The standard deviation in the molecular weight distribution (MWD) is required in order to determine the maximum error associated with the mean aggregation number for the spheres and vesicles (i.e., N_s and N_v, respectively). This is because the dominant error in this calculation comes from the uncertainty in the mean volume occupied by one core-forming PHPMA block, which in turn is determined by the MWD of the $PGMA_{45}$-$PHPMA_{200}$ diblock copolymer. Therefore, the MWD determined by DMF GPC analysis of the laboratory-scale synthesis (see the black trace shown in Figure S2) was fitted to a Gaussian model (see blue trace in Figure S2) to estimate this standard deviation using Equation S3, as shown below:

$$y = a\,exp\left(-\frac{(x-b)^2}{2\sigma^2}\right) \tag{S3}$$

Here a and b are constants, y is the retention time, x is the detector response and σ is the standard deviation. This σ value corresponded to 6% of the peak retention time for the $PGMA_{45}$-$PHPMA_{200}$ diblock copolymer. This parameter was subsequently used as the maximum percentage error for the relevant N_s (or N_v) calculations.

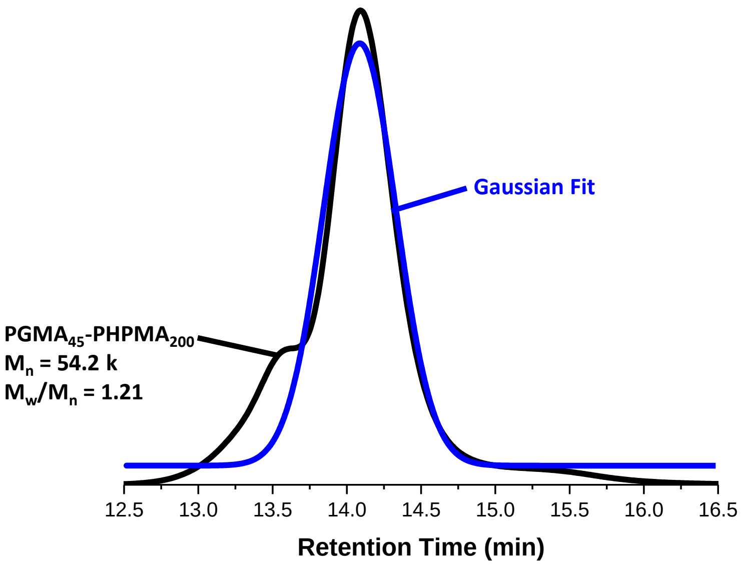

Figure S2. DMF gel permeation chromatogram (black trace) obtained for the final $PGMA_{45}$-$PHPMA_{200}$ diblock copolymer chains produced by the laboratory-scale RAFT aqueous dispersion polymerization of HPMA at 70 °C targeting 10% w/w solids. The blue trace depicts a Gaussian fit to the black trace.

2.5 N_{agg}, S_{agg}, and d_{int} equations

It is possible to calculate the mean number of copolymer chains per nano-object (N_s, N_w or N_v for spheres, worms or vesicles; generically denoted herein as N_{agg}), the mean number of copolymer chains per unit area (S_{agg}), and the average distance between two copolymer chains at the core-shell interface (d_{int}). The corresponding equations are given below with explanatory cartoons provided where required.

Spheres

$$N_s = (1 - \varphi_{sol}) \text{ x } \frac{\frac{4}{3}\pi R_c^3}{V_c}$$

$$S_{agg} = \frac{N_s}{4\pi R_s^2}$$

$$d_{int} = \sqrt{\frac{1}{S_{agg}}} = \sqrt{\frac{4\pi R_s^2}{N_s}}$$

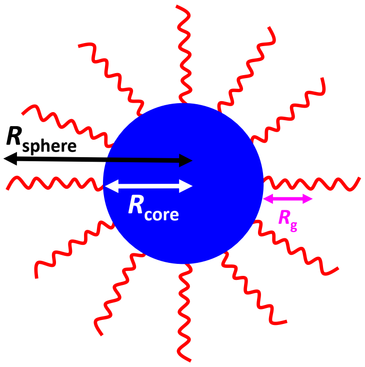

where φ_{sol} is the solvent volume fraction within the spherical cores, R_c is the core radius, V_c is the volume of the core [see Equation S2] and R_s is the sphere radius (i.e. $R_s = R_c + 2.R_g$).

Worms

$$N_w = (1 - \varphi_{sol}) \text{ x } \frac{\pi R_c^3 L_w}{V_c}$$

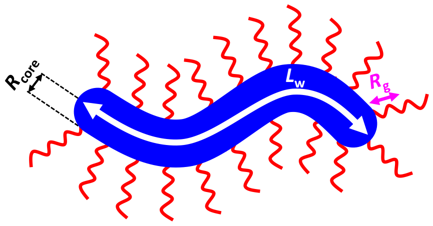

where φ_{sol} is the volume fraction of solvent in the core, R_c is the worm core cross-sectional radius, L_w is the mean worm length and V_c is the worm core volume [see Equation S2].

Vesicles

$$N_v = (1 - \varphi_{sol}) \text{ x } \frac{V_{out} - V_{in}}{V_m}$$

$$S_{agg} = \frac{N_v}{4\pi({R_{out}}^2 + {R_{in}}^2)}$$

$$d_{int} = \sqrt{\frac{1}{S_{agg}}} = \sqrt{\frac{4\pi({R_{out}}^2 + {R_{in}}^2)}{N_v}}$$

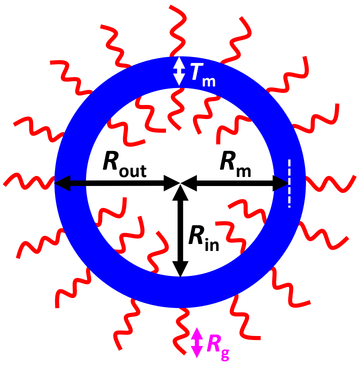

R_m = Radius from centre of vesicle to middle of membrane

$R_{out} = R_m + ½T_m$
$R_{in} = R_m - ½T_m$

where φ_{sol} is the solvent volume fraction within the membrane, T_m is the vesicle membrane thickness and V_m is the membrane volume [see Equation S2].

$$V_{out} = \frac{4}{3}\pi R_{out}^3 \qquad V_{in} = \frac{4}{3}\pi R_{in}^3 \qquad R_{out} = R_m + \frac{1}{2}T_m \qquad R_{in} = R_m - \frac{1}{2}T_m$$

3. Supporting Figures

3.1 Micellar Nucleation

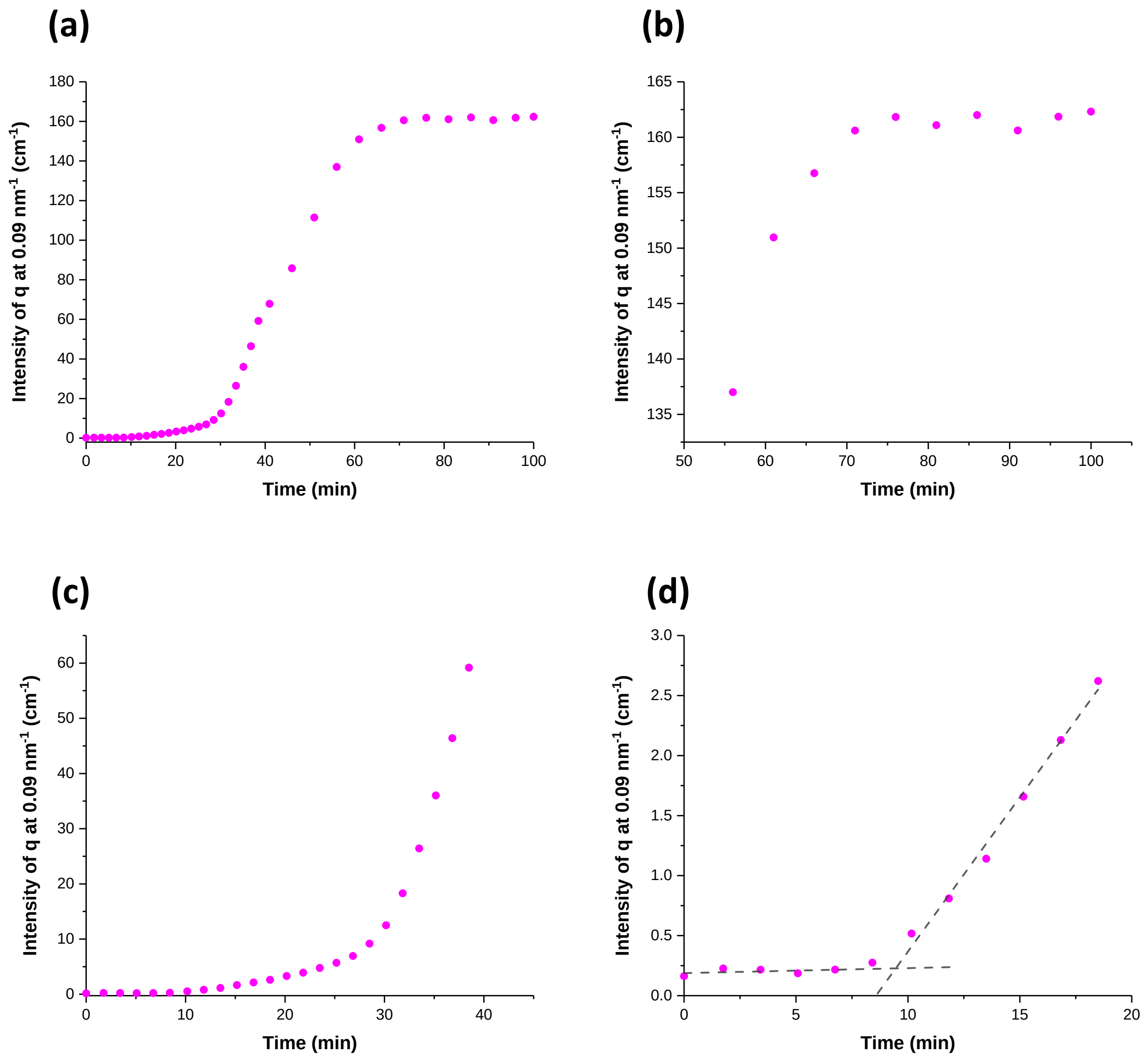

Figure S3. (a) Scattered X-ray intensity $I(q)$ at 0.09 nm^{-1} recorded throughout the HPMA polymerization when targeting $PGMA_{45}$-$PHPMA_{200}$ vesicles at 70 ◦C and 10% w/w solids; (b) $I(q)$ recorded at an arbitrary q value of 0.09 nm^{-1} towards the end of polymerization, with the plateau at 76 min corresponding to the end of the polymerization; (c) $I(q)$ at 0.09 nm^{-1} for the first 40 min of polymerization showing the pronounced upturn in scattered X-ray intensity that is observed after 30 min, and (d) $I(q)$ at 0.09 nm^{-1} for the first 18 min of polymerization highlighting an upturn in intensity at 10 min.

3.2 Data Fits to Selected SAXS patterns

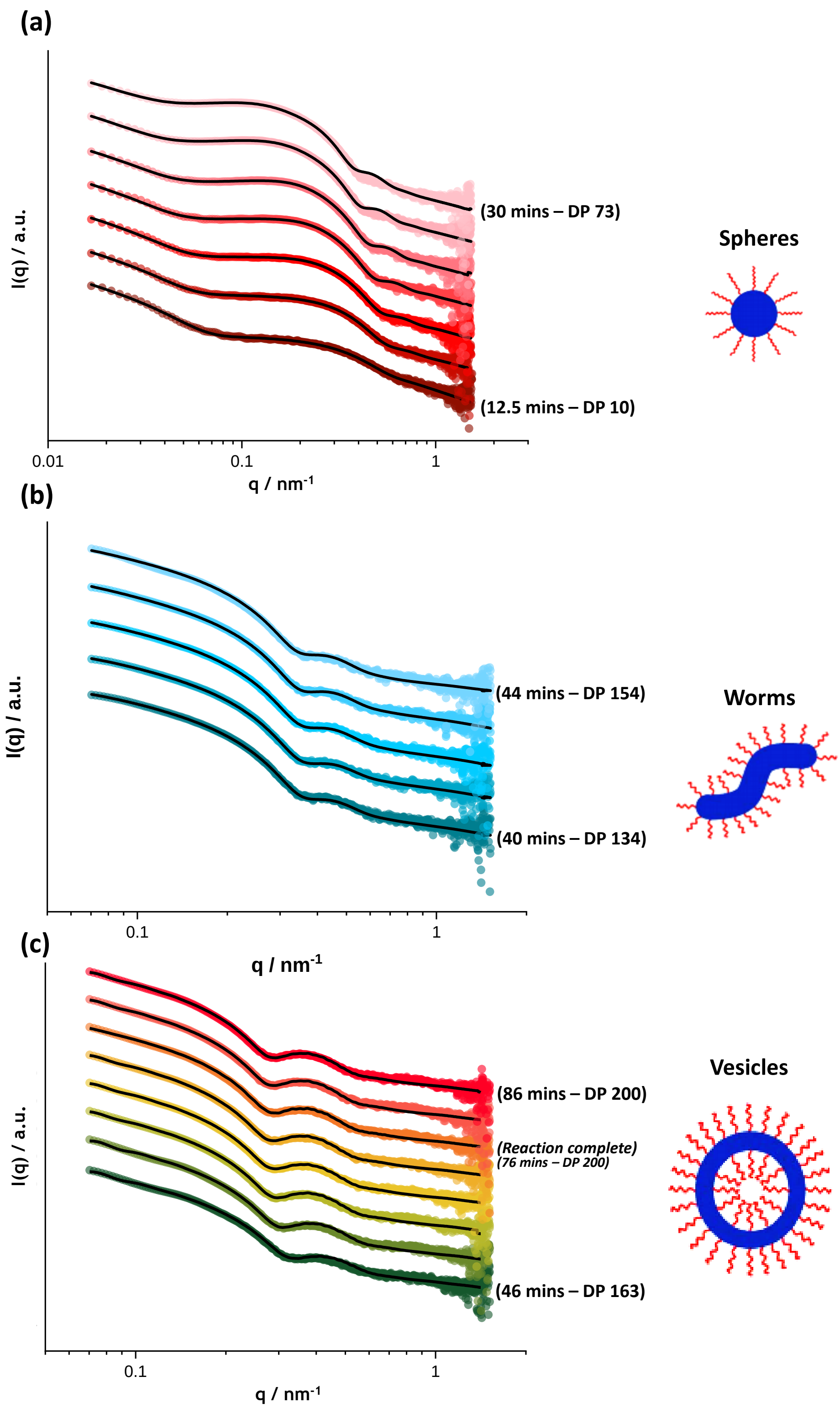

Figure S4. Data fits to the SAXS patterns recorded for each copolymer morphology during the *in situ* SAXS experiment: (a) spheres, (b) worms and (c) vesicles. Corresponding reaction times and instantaneous PHPMA DPs for the first and last SAXS pattern in each case.

3.3 TEM images for spheres/worms, worms and nascent vesicles

(a) 35 min

(b) 40 min

(c) 45 min

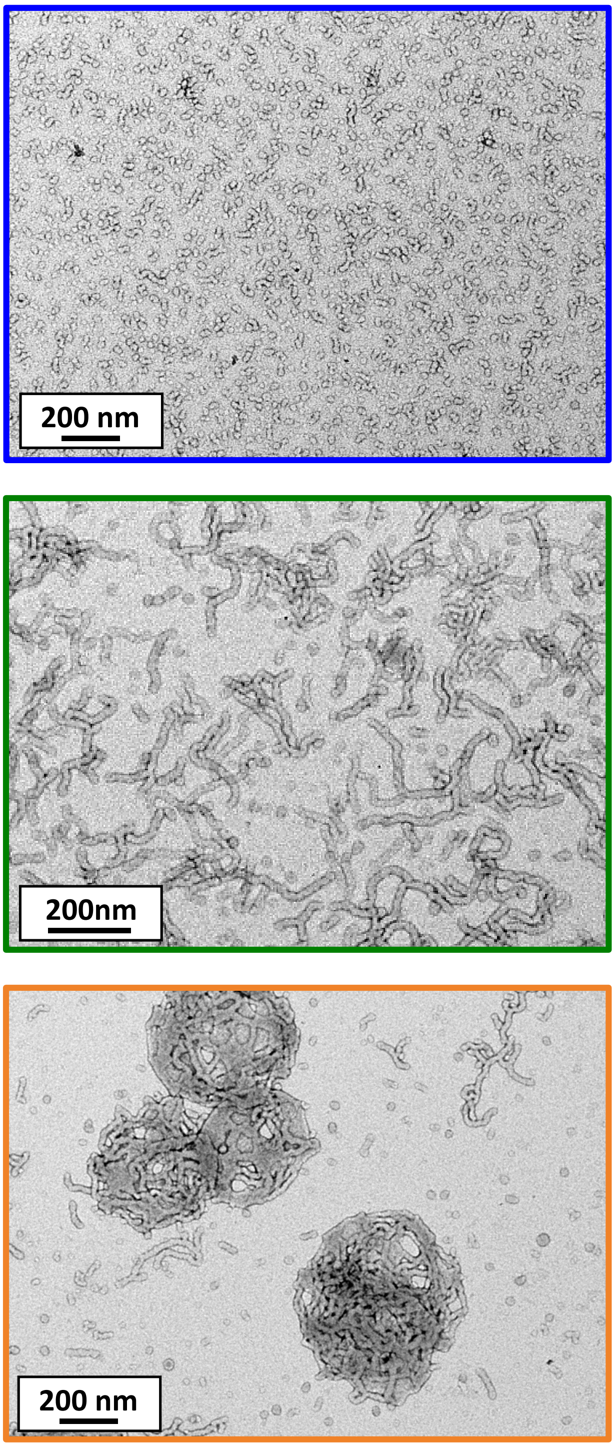

Figure S5. TEM images (and corresponding reaction times) recorded during the laboratory-based synthesis of $PGMA_{45}$-$PHPMA_{200}$ vesicles at 70 ◦C depicting (a) spheres and initial worm formation, (b) worm growth, and (c) nascent vesicle formation.

3.4 TEM images of transient intermediate structures

(a)

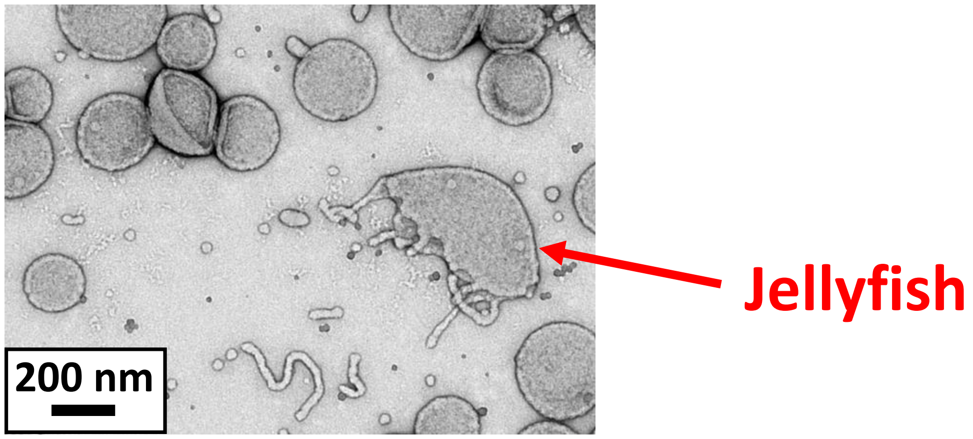

(b)

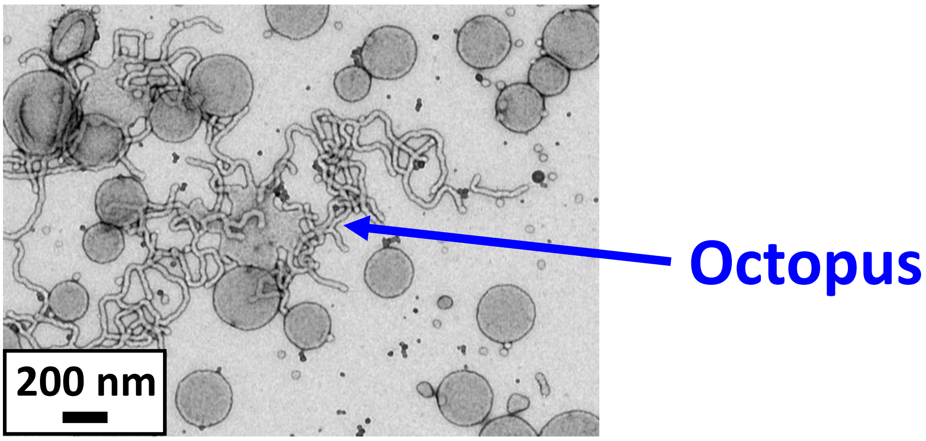

Figure S6. TEM images recorded at intermediate conversions during the laboratory-based synthesis of $PGMA_{45}$-$PHPMA_{200}$ vesicles showing the transient intermediate structures observed during the transformation of worms into vesicles: (a) jellyfish and (b) octopus.

3.5 TEM image of the final vesicles

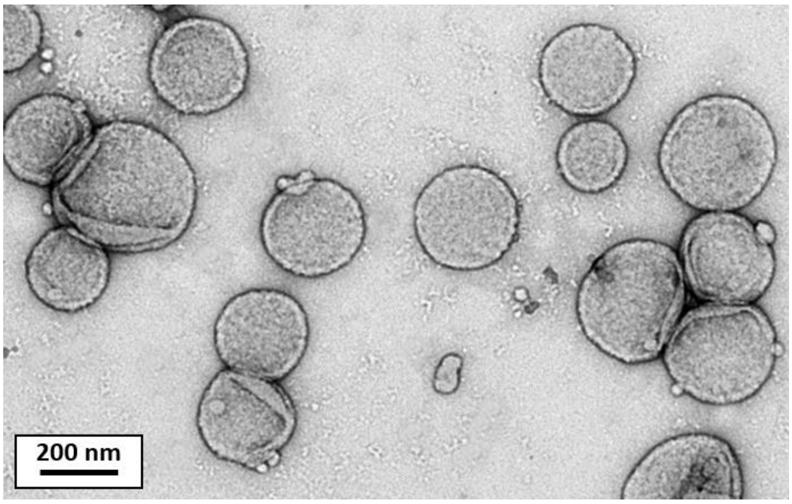

Figure S7. *Postmortem* TEM image recorded after the *in situ* SAXS experiment conducted using the stirrable SAXS cell at 70 °C while targeting $PGMA_{45}$-$PHPMA_{200}$ vesicles at 10% w/w solids. The final copolymer morphology corresponds to polydisperse vesicles.

3.6 Comparison of GPC data obtained for the laboratory-based synthesis and the *in situ* SAXS experiment

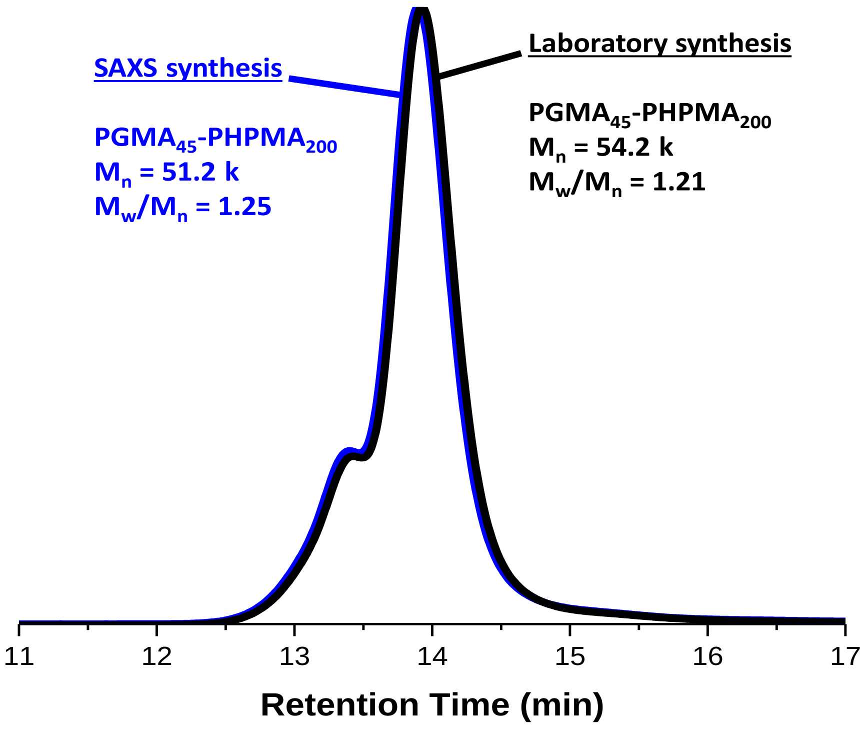

Figure S8. DMF GPC traces for the final reaction mixture recorded after (i) the laboratory-based synthesis (black line) and (ii) the *in situ* SAXS experiment (blue line) when targeting $PGMA_{45}$-$PHPMA_{200}$ vesicles at 70 °C and 10% w/w solids. Clearly, there are minimal differences between these two GPC traces, indicating that essentially the same copolymer chains are obtained in each case.

4. SAXS Models

In general, the intensity of X-rays scattered by a dispersion of nano-objects [usually represented by the scattering cross-section per unit sample volume, $\frac{d\Sigma}{d\Omega}(q)$] can be expressed as:

$$\frac{d\Sigma}{d\Omega}(q) = NS(q) \int_0^\infty \dots \int_0^\infty F(q, r_1, \dots, r_k)^2 \Psi(r_1, \dots, r_k) dr_1, \dots, dr_k \quad \text{(S4)}$$

where $F(q, r_1, \dots, r_k)$ is the form factor, $r_1, \dots, r_k$ is a set of k parameters describing the structural morphology, $\Psi(r_1, \dots, r_k)$ is the distribution function, $S(q)$ is the structure factor and N is the nano-object number density per unit volume expressed as:

$$N = \frac{\varphi}{\int_0^\infty \dots \int_0^\infty V(r_1, \dots, r_k) \Psi(r_1, \dots, r_k) dr_1, \dots, dr_k} \quad \text{(S5)}$$

where $V(r_1, \dots, r_k)$ is the volume of the nano-object and φ is the volume fraction of nano-objects.

4.1 Spherical micelle model

The spherical micelle form factor for Equation S4 is given by:[4]

$$F_{s_{mic}}(q) = N_s^2 \beta_s^2 A_s^2(q, R_s) + N_s \beta_c^2 F_c(q, R_g) + N_s(N_s - 1)\beta_c^2 A_c^2(q) + 2N_s^2 \beta_c A_s(q, R_s) A_c(q) \quad \text{(S6)}$$

where R_s is the core radius of the spherical micelle, R_g is the radius of gyration of the PGMA corona block. The X-ray scattering length contrast for the core block and the corona block is given by $\beta_s = V_s(\xi_s - \xi_{sol})$ and $\beta_c = V_c(\xi_c - \xi_{sol})$, respectively. Here ξ_s, ξ_c and ξ_{sol} are the X-ray scattering length densities of the core block (ξ_{PHPMA} = 11.11 × 10^{10} cm^{-2}), the corona block (ξ_{PGMA} = 11.94 × 10^{10} cm^{-2}) and the solvent (ξ_{sol} = 9.42 × 10^{10} cm^{-2}), respectively. V_s and V_c are the volumes of the core block (V_{PHPMA}) and the corona block ($V_{PGMA_{45}}$), respectively. These volumes were calculated using Equation S2, see Section 2.2. The sphere form factor amplitude is used for the amplitude of the core self-term:

$$A_c(q, R_s) = \Phi(qR_s) exp\left(-\frac{q^2\sigma^2}{2}\right) \quad \text{(S7)}$$

where $\Phi(qR_{\mathrm{s}}) = \frac{3[\sin(qR_{\mathrm{s}}) - qR_{\mathrm{s}}\cos(qR_{\mathrm{s}})}{(qR_{\mathrm{s}})^3}$. A sigmoidal interface between the two blocks was assumed for the spherical micelle form factor. This is described by the exponent term with a width σ accounting for a decaying scattering length density at the core-shell interface of the deblock copolymer micelle (or nanoparticle). This σ value was fixed at 2.5 during fitting.

The form factor amplitude of the spherical micelle corona is:

$$A_{\mathrm{c}}(q) = \frac{\int_{R_{\mathrm{s}}}^{R_{\mathrm{s}}+2s} \mu_{\mathrm{c}}(r)\frac{\sin(qr)}{qr} r^2 dr}{\int_{R_{\mathrm{s}}}^{R_{\mathrm{s}}+2s} \mu_{\mathrm{c}}(r) r^2 dr} \exp\left(-\frac{q^2\sigma^2}{2}\right) \quad \text{(S8)}$$

The radial profile, $\mu_{\mathrm{c}}(r)$, can be expressed by a linear combination of two cubic b splines, with two fitting parameters s and a that correspond to the width of the profile and the weight coefficient, respectively. The self-correlation term for the coronal block is given by the Debye function:

$$F_{\mathrm{c}}(q, R_{\mathrm{g}}) = \frac{2[\exp(-q^2R_g^2) - 1 + q^2R_{\mathrm{g}}^2]}{q^4R_{\mathrm{g}}^4} \quad \text{(S9)}$$

where R_{g} is the radius of gyration of the PGMA coronal block. The aggregation number, N_{s}, for the spherical micelle is given by:

$$N_{\mathrm{s}} = (1 - x_{\mathrm{sol}})\frac{\frac{4}{3}\pi R_S^3}{V_S} \quad \text{(S10)}$$

where x_{sol} is the volume fraction of solvent within the micelle cores (formed by the PHPMA block in the present case). An effective structure factor expression proposed for interacting micelles[5] has been used in Equation S4 to give:

$$S_{\mathrm{s}}(q) = 1 + \frac{A_{\mathrm{s_{mic}}}^{\mathrm{av}}(q)^2[S_{\mathrm{PY}}(q, R_{\mathrm{PY}}, f_{\mathrm{PY}}) - 1}{F_{\mathrm{s_{mic}}}(q)} \quad \text{(S11)}$$

Herein the form factor of the average radial scattering length density distribution of micelles is used as $A_{\mathrm{s_{mic}}}^{\mathrm{av}}(q) = N_{\mathrm{s}}[\beta_{\mathrm{s}}A_{\mathrm{s}}(q, R_{\mathrm{s}}) + \beta_{\mathrm{c}}A_{\mathrm{c}}(q)]$ and $S_{\mathrm{PY}}(q, R_{\mathrm{PY}}, f_{\mathrm{PY}})$ is a structure factor for hard-sphere interactions based on the Percus-Yevick approximation,[6] where R_{PY} is the interaction radius and f_{PY} is the hard-sphere volume fraction. The micelle model assumes a polydispersity for one parameter (R_{s}), which can be described by a Gaussian distribution. Thus, the polydispersity function in Equation S4 can be represented as:

$$\Psi(r_1) = \frac{1}{\sqrt{2\pi\sigma_{R_S}{}^2}} \exp\left(-\frac{(r_1 - R_\mathrm{S})^2}{2\sigma_{R_S}{}^2}\right) \tag{S12}$$

where σ_{Rs} is the standard deviation for R_s. In accordance with Equation S5, the number density per unit volume for the micelle is expressed as:

$$N = \frac{\varphi}{\int_0^\infty V(r_1)\Psi(r_1)dr_1} \tag{S13}$$

Here φ is the copolymer volume fraction and $V(r_1)$ is the total copolymer volume in a spherical micelle $[V(r_1) = (V_\mathrm{s} + V_\mathrm{c})N_\mathrm{s}(r_1)]$.

4.2 Worm-like micelle model

According to Equation S4, the form factor for a worm-like micelle can be expressed as:

$$F_{\mathrm{w_{mic}}}(q) = N_\mathrm{w}^2\beta_\mathrm{s}^2F_\mathrm{w}(q) + N_\mathrm{w}\beta_c^2F_\mathrm{c}(q, R_g) + N_\mathrm{w}(N_\mathrm{w} - 1)\beta_c^2S_\mathrm{cc}(q) + 2N_\mathrm{w}^2\beta_s\beta_cS_\mathrm{sc}(q) \tag{S14}$$

Unless stated otherwise, all parameters are the same as those described in the spherical micelle model (see Equation S6).

The self-correlation term for the cross-sectional volume-average radius R_w within the worm core is:

$$F_w(q) = F_\mathrm{worm}(q, L_\mathrm{w}, b_\mathrm{w})A^2_{\mathrm{cs_{worm}}}(q, R_\mathrm{w}) \tag{S15}$$

where

$$A^2_{\mathrm{cs_{worm}}}(q, R_\mathrm{w}) = \left[2\frac{J_1(qR_\mathrm{w})}{qR_\mathrm{w}}\right]^2 \tag{S16}$$

Here J_1 is the first-order Bessel function of the first kind, and a form factor $F_\mathrm{worm}(q, L_\mathrm{w}, b_\mathrm{w})$ for self-avoiding semi-flexible chains represents the worm-like micelles, where b_w is the Kuhn length and L_w is the mean contour length. A full expression for the chain form factor can be found elsewhere.[7]

The mean aggregation number for the worm-like micelle, N_w, is given by:

$$N_\mathrm{w} = (1 - x_\mathrm{sol})\frac{\pi R_\mathrm{w}^2 L_\mathrm{w}}{V_s} \tag{S17}$$

where x_{sol} is the volume fraction of solvent within the worm-like micelle core. The possible presence of semi-spherical caps at the two ends of each worm is neglected for this form factor.

A polydispersity for one parameter (R_{w}) is assumed for this worm-like micelle model, which can be described by a Gaussian distribution. Thus, the polydispersity function in Equation S4 can be represented as:

$$\Psi(r_1) = \frac{1}{\sqrt{2\pi\sigma_{R_W}{}^2}} \exp\left(-\frac{(r_1 - R_{\text{w}})^2}{2\sigma_{R_W}{}^2}\right) \tag{S18}$$

Here σ_{R_W} is the standard deviation for R_{w}. In accordance with Equation S5, the number density per unit volume for this worm-like micelle model is expressed as:

$$N = \frac{\varphi}{\int_0^{\infty} V(r_1)\Psi(r_1)dr_1} \tag{S19}$$

Here φ is the copolymer volume fraction and $V(r_1)$ is the total copolymer volume in a worm-like micelle $[V(r_1) = (V_{\text{s}} + V_{\text{c}})N_{\text{w}}(r_1)]$.

4.3 Vesicle model

The vesicle form factor in Equation S4 is expressed as:[8]

$$F_{\text{vmic}}(q) = \mathrm{N_v^2 \beta_s^2 A_m^2(q) + N_v \beta_c^2 F_c(q, R_g) + N_v(N_v - 1)\beta_c^2 A_{vc}^2(q) + 2N_v^2 \beta_c} \beta_{\mathrm{s}} \mathrm{A_m(q) A_{vc}(q)} \tag{S20}$$

Unless stated otherwise, all parameters are the same as those defined in the spherical micelles model (Equation S6).

The amplitude of the membrane self-term is:

$$A_{\text{m}}(q) = \frac{V_{\text{out}}\varphi(qR_{\text{out}}) - V_{\text{in}}\varphi(qR_{\text{in}})}{V_{\text{out}} - V_{\text{in}}} \exp\left(-\frac{q^2\sigma_{\text{in}}{}^2}{2}\right) \tag{S21}$$

where $R_{\text{in}} = R_{\text{m}} - \frac{1}{2}T_{\text{m}}$ is the inner radius of the membrane and $R_{\text{out}} = R_{\text{m}} + \frac{1}{2}T_{\text{m}}$ is the outer radius of the membrane (R_{m} is the radius from the centre of the vesicle to the centre of the membrane), and $V_{\text{in}} = \frac{4}{3}\pi R_{\text{in}}{}^3$ and $V_{\text{out}} = \frac{4}{3}\pi R_{\text{out}}{}^3$. The exponent term in Equation S21 represents a sigmoidal interface between the two blocks, with a width σ_{in} accounting for a decaying scattering length density at the membrane surface. The value of σ_{in} was fixed at 2.5 during fitting. The mean vesicle aggregation number, N_{v}, is given by:

$$N_{\mathrm{v}} = (1 - x_{\mathrm{sol}})\frac{V_{\mathrm{out}} - V_{\mathrm{in}}}{V_{\mathrm{s}}} \tag{S22}$$

where x_{sol} is the solvent volume fraction within the vesicle membrane. Assuming that there is no penetration of the hydrophilic coronal blocks into the hydrophobic membrane, the amplitude of the vesicle corona self-term is expressed as:

$$A_{\mathrm{vc}}(q) = \Psi(qR_{\mathrm{g}})\frac{1}{2}\left[\frac{\sin\,[q(R_{\mathrm{out}}+R_{\mathrm{g}})]}{q(R_{\mathrm{out}}+R_{\mathrm{g}})} + \frac{\sin\,[q(R_{\mathrm{in}}-R_{\mathrm{g}})]}{q(R_{\mathrm{in}}-R_{\mathrm{g}})}\right] \tag{S23}$$

where the term outside the square brackets is the form factor amplitude of the corona block such that:

$$\Psi(qR_{\mathrm{g}}) = \frac{1-\exp\,(-\mathrm{q}R_{\mathrm{g}})}{(qR_{\mathrm{g}})^2} \tag{S24}$$

For the vesicle model, it is assumed that two parameters are polydisperse: the radius from the centre of the vesicle to the centre of the membrane (R_{m}) and the membrane thickness (T_{m}). Each parameter is considered to have a Gaussian distribution, hence the polydispersity function in Equation S4 can be expressed in each case as:

$$\Psi(r_1 r_2) = \frac{1}{\sqrt{2\pi\sigma_{R_S}{}^2}}\exp\left(-\frac{(r_1-R_{\mathrm{m}})^2}{2\sigma_{R_m}{}^2}\right)\frac{1}{\sqrt{2\pi\sigma_{T_{\mathrm{m}}}{}^2}}\exp\left(-\frac{(r_1-T_{\mathrm{m}})^2}{2\sigma_{T_m}{}^2}\right) \tag{S25}$$

Where $\sigma_{R_{\mathrm{m}}}$and $\sigma_{T_{\mathrm{m}}}$are the standard deviations for R_{m} and T_{m}, respectively. According to Equation S5, the number density per unit volume for the vesicle model can be expressed as:

$$N = \frac{\varphi}{\int_0^{\infty}\int_0^{\infty} V(r_1,r_2)\Psi(r_1,r_2)dr_1 dr_2} \tag{S26}$$

Here φ is the copolymer volume fraction and $V(r_1, r_2)$ is the total copolymer volume $[V(r_1, r_2) = (V_{\mathrm{s}} + V_{\mathrm{c}})N_v(r_1, r_2)]$.

Programming tools within the Irena SAS Igor Pro macros[9] were used to implement the scattering models.

References

1 M. Semsarilar, V. Ladmiral, A. Blanazs and S. P. Armes, *Langmuir*, 2012, **28**, 914–922.

2 N. J. Warren, O. O. Mykhaylyk, A. J. Ryan, M. Williams, T. Doussineau, P. Dugourd, R. Antoine, G. Portale and S. P. Armes, *J. Am. Chem. Soc.*, 2015, **137**, 1929–1937.

3 L. J. Fetters, D. J. Lohse and R. H. Colby, *Chain Dimensions and Entanglement Spacings*, Springer, New York NY, New York, NY, 2007.

4 J. S. Pedersen, *J. Appl. Crystallogr.*, 2000, **33**, 637–640.

5 J. S. Pedersen, *J. Chem. Phys.*, 2001, **114**, 2839–2846.

6 D. J. Kinning and E. L. Thomas, *Macromolecules*, 1984, **17**, 1712–1718.

7 J. S. Pedersen and P. Schurtenberger, *Macromolecules*, 1996, **29**, 7602–7612.

8 J. Bang, S. Jain, Z. Li, T. P. Lodge, J. S. Pedersen, E. Kesselman and Y. Talmon, *Macromolecules*, 2006, **39**, 1199–1208.

9 J. Ilavsky and P. R. Jemian, *J. Appl. Crystallogr.*, 2009, **42**, 347–353.
